# Reducing the carbon footprint of diets across socio-demographic groups in Finland: a mathematical optimisation study

**DOI:** 10.1017/S1368980024000508

**Published:** 2024-03-04

**Authors:** Xavier Irz, Heli Tapanainen, Merja Saarinen, Jani Salminen, Laura Sares-Jäske, Liisa M Valsta

**Affiliations:** 1 Department of Economics and Management, University of Helsinki, Latokartanonkaari 5, Helsinki, Finland; 2 Bioeconomy Policies and Markets Group, Natural Resources Institute Finland, Latokartanonkaari 9, PL 2, Helsinki, Finland; 3 Department of Public Health and Welfare, Finnish Institute for Health and Welfare, Helsinki, Finland; 4 Sustainability Science and Indicators Group, Natural Resources Institute Finland, Latokartanonkaari 9, PL 2, Helsinki, Finland; 5 Finnish Environment Institute, Latokartanonkaari 11, Helsinki, Finland

**Keywords:** Diet, Food consumption, Optimisation, Sustainability, Climate change, Environmental impact, Just transition

## Abstract

**Objectives:**

To characterise nutritionally adequate, climate-friendly diets that are culturally acceptable across socio-demographic groups. To identify potential equity issues linked to more climate-friendly and nutritionally adequate dietary changes.

**Design:**

An optimisation model minimises distance from observed diets subject to nutritional, greenhouse gas emissions (GHGE) and food-habit constraints. It is calibrated to socio-demographic groups differentiated by sex, education and income levels using dietary intake data. The environmental coefficients are derived from life cycle analysis and an environmentally extended input–output model.

**Setting:**

Finland.

**Participants:**

Adult population.

**Results:**

Across all population groups, we find large synergies between improvements in nutritional adequacy and reductions in GHGE, set at one-third or half of the current level. Those reductions result mainly from the substitution of meat with cereals, potatoes and roots and the intra-category substitution of foods, such as beef with poultry in the meat category. The simulated more climate-friendly diets are thus flexitarian. Moving towards reduced-impact diets would not create major inadequacies related to protein and fatty acid intakes, but Fe could be an issue for pre-menopausal females. The initial socio-economic gradient in the GHGE of diets is small, and the patterns of adjustments to more climate-friendly diets are similar across socio-demographic groups.

**Conclusions:**

A one-third reduction in GHGE of diets is achievable through moderate behavioural adjustments, but achieving larger reductions may be difficult. The required changes are similar across socio-demographic groups and do not raise equity issues. A population-wide policy to promote behavioural change for diet sustainability would be appropriate.

Recent research has produced a strong scientific consensus that the global food system is fundamentally unsustainable as it operates beyond planetary boundaries^(1)^ and produces negative nutritional outcomes^(2)^ that may worsen in the face of population growth over the coming decades. The need for systemic reforms to achieve sustainability is encapsulated by the EAT-Lancet Commission’s call for a ‘Great Food Transformation’^(3)^, which has resulted in high-level policy initiatives such as the 2021 UN Food System Summit^(4)^, or the food system component of the European Union’s Farm to Fork strategy^(5)^.

Population-level dietary change forms a central pillar of the advocated transformation, as there is strong evidence that the environmental impacts of foods vary enormously and that lower-impact diets can be compatible with healthiness^(6)^. The search for sustainable diets has therefore received much attention in recent years. At a general level, those are defined as the ‘dietary patterns that promote all dimensions of individuals’ health and wellbeing; have low environmental pressure and impact; are accessible, affordable, safe and equitable; and are culturally acceptable’^(2)^. Although appealing at a conceptual level, this definition is too general to support policy actions. Consequently, there is a need to characterise sustainable diets much more precisely, in particular in terms of their detailed ingredient composition.

However, the practical identification of sustainable diets raises a number of challenges that have only been partially addressed in existing literature^(7)^. The first difficulty lies with the near-infinite number of food combinations that could be deemed sustainable, so a trial-and-error approach to the search for sustainable diets, while useful, is likely to generate sub-optimal solutions and be strongly influenced by the researcher’s prior beliefs as well as commonly accepted dietary patterns. A more systematic and general approach to the problem of identifying sustainable diets is therefore called for. A second issue relates to the difficulty of operationalising some qualitative concepts, such as cultural acceptability, in the analysis. While there is ample evidence that food consumption is highly influenced by social and cultural factors^(8)^, few practical tools are available to compare the acceptability of alternative diets, as reviewed by Gazan *et al.* (2016)^(7)^, although we acknowledge recent developments^(9)^. The strong sociocultural dimension of diets, however, implies at a minimum that dietary changes for sustainability should be investigated in varying national and regional contexts^(10)^. Finally, although the above-cited definition of sustainable diets makes explicit mention of equity issues, those have not been included in empirical investigations beyond the analysis of affordability in some rare cases^(11)^.

This paper presents a diet optimisation model, which identifies combinations of foods that meet a detailed list of nutritional recommendations^(12,13)^, remain as similar as possible to existing diets in Finland and have lower overall greenhouse gas emissions (GHGE). A specificity is that the model is calibrated to different socio-demographic groups of the Finnish adult population to measure the extent to which the dietary changes necessary to reduce GHGE vary along well-defined socio-demographic lines. That question has not been investigated previously, although it has important policy implications. If more climate-friendly dietary changes vary considerably across sub-populations, targeted policies as opposed to population-wide ones would be preferable, for instance, when communicating the nature of the foods whose consumption should increase or decrease. The research also aims at identifying population groups for which the transition towards more climate-friendly diets could be particularly difficult and pose equity issues. This will help identify potential political obstacles to the implementation of policies for dietary changes and consider the need for accompanying measures targeted at specific and vulnerable sub-populations.

## Methods

### The diet optimisation model

The model identifies diets that minimise the sum of squared relative deviations from the observed average diet of different socio-demographic groups, subject to a set of nutritional, food-habit, GHGE and food system constraints, which together ensure the nutritional adequacy, acceptability and reduced GHGE of the solution diet. Socio-demographic groups are defined based on sex, education level and income level, as explained in the data section. The full mathematical presentation of the model is found in Appendix B, as we only outline its main characteristics here. Formally, the objective function is 
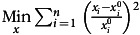
 , where ***x*** denotes an *n*-vector of average consumption *x*_*i*_ of each food *i*, and 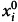 defines the observed (=current) average consumption of food *i* in each socio-demographic group of interest. The procedure limits departure from the observed average diet subject to the constraints and by doing so maximises the cultural acceptability and achievability of the simulated dietary changes. The implicit idea considers that observed diets already embed consumer preferences and the difficult trade-offs involved in food choices. Hence, radical changes from observed choices may be difficult to achieve in the short term in most situations^(14)^. This general line of reasoning has been used previously in many published studies on diet optimisation that minimise deviation from observed diets^(7,9)^.

A first linear constraint imposes the constancy of energy intake, which is set at its observed level in the dietary intake data. Thus, all simulations are isoenergetic, and we abstract from addressing the relevant but different issue of optimal energy intake in order to focus solely on that of diet composition.

A set of constraints defines the minimum for recommended^(12,13)^ or safe^(15)^ daily intake and the maximum for recommended daily intake or upper level for safe intake for a detailed list of macronutrients (*n* 30), vitamins (*n* 13) and minerals (*n* 18) listed in Appendix A, Table A.1. The values were drawn from the Nordic Nutrition Recommendations 2012^(12)^, Finnish Nutrition Recommendations 2014^(13)^ and for amino acids from the WHO’s protein and amino acid recommendation^(15)^, namely, individual amino acid requirement with added 24 % safety margin. This was a slightly more conservative approach than using the average requirement reference values. This approach was chosen due to the fact that the data used in this study did not represent the usual intake of the population groups but were group averages and thus did not fulfil the prerequisites for using the average requirement values as a reference. There was, though, one exception in using the recommended daily intake type of reference value for the Fe constraint, as previous research has shown that dietary Fe intake is not associated with Fe status among pre-menopausal Finnish women^(16,17)^. Fe status among these women is mainly affected by blood losses. For that population group, it is difficult to improve Fe status by increasing dietary intakes only, and reaching the recommended daily intake requires other changes, such as Fe fortification and Fe supplementation that were not included in the analysis. In order not to constrain the model unnecessarily, the minimum Fe intake for women was therefore set to its level observed in the Finnish diet, which meets the recommended daily intake of post-menopausal women but only the average Fe requirement in case of pre-menopausal women^(12,18)^. The importance of that assumption is analysed further in the sensitivity analysis. The detailed list of recommended or safe daily intakes makes clear that the adequacy of protein, fatty acid and carbohydrate intakes is explicitly taken into account in the analysis. Imposition of those constraints ensures that all the solution diets are, by construction, nutritionally adequate according to the selected set of nutritional criteria.

A set of food-habit constraints also imposes that the optimal consumption of any food category should be no less than the 10th centile of the consumption distribution of that food in the sub-population of interest and no more than the 90th centile, following the assumptions of Vieux *et al.* (2018)^(19)^. This prevents the solution diets from including the consumption of some foods at levels that are not observed in the population of interest, hence reinforcing cultural acceptability beyond what is captured through the objective function.

A single environmental constraint sets an exogenously given maximum level of GHGE from the diet (see section “Scenarios”). Finally, a constraint is introduced to reflect the jointness of dairy and beef production in the Finnish food system^(20)^: at present, the beef-to-dairy ratio cannot realistically fall under a minimum level as roughly 80 % of beef in Finland originates from the dairy chain. The study of the Dutch diet by Broekema *et al.* (2020)^(21)^ introduces a similar constraint. We estimated that, from the Finnish dairy chain, for each gram of beef carcass, 33·9 g of raw milk are produced. The beef content of the relevant food ingredients (in parentheses) was also estimated to quantify the ratio of raw milk to beef production: beef (100%), offals (88%), meat products (50%), sausages (7·5%), sausage cuts (7·5%) and meat cuts (7·5%).

The above structure defines a classic quadratic programming problem, in which a quadratic objective function is minimised subject to a set of linear equality and inequality constraints. Although the numerical solutions to those types of problems can be local rather than global, the exact form of our objective function ensures that this is not an issue here as explained further in Appendix B. Thus, the numerical optimisation derived by applying the *R* package quadprog^(22)^ gives the global solution to the diet optimisation problem.

### Data

#### Dietary intakes and food composition

The National FinDiet 2017 Survey^(23)^ provided a detailed description of the average diet of various sub-groups of the Finnish adult population differentiated by sex, income quintile and educational level. The nationally representative FinDiet 2017 survey is a subsample (*n* 3099) of the FinHealth 2017 Study (*n* 10 247)^(24)^. This analysis used data from 1655 adults aged 18–74 years (875 females and 780 males, 53 % of the invited) with two non-consecutive 24-h dietary recalls. The in-house dietary software Finessi (THL, Finland) and the National Food Composition Database Fineli® (FCDB) were used to calculate the nutrient intakes of different diets*. Food consumption was estimated at the ingredient level after disaggregating the consumed foods according to the recipes of the FCDB. The nutrient composition of a food category was derived by calculating the weighted sum of nutrient intakes of all food items belonging to the food category. The weights for every food item were calculated as the share of the consumption of a food item from the consumption of the whole food category in the FinDiet 2017 Survey data. The model was built on a food categorisation incorporated in the FCDB. Some categories were aggregated for this analysis, but the final classification (seventy-four food categories) elaborated by nutritionists was kept sufficiently disaggregated to allow for precise nutritional and climate impact assessments. In some cases, these seventy-four food categories were aggregated after completion of the optimisation process into thirteen main food categories to facilitate reporting and analysis.

#### Background information and socio-demographic groups

Self-reported total years of education were categorised into tertiles (low, medium, and high) according to sex and birth year. The income quintile was based on questions on total household income during the previous year before tax deductions and on the number of adult and underage household members. The groups included in the analysis for each sex were the whole adult population, all three educational tertiles and three income quintiles (1st, 3rd and 5th).

The GHGE coefficients were generated using Life Cycle Assessment (LCA) as presented in Saarinen *et al.* (2019)^(25)^. The coefficients are reported in Appendix A, Table A.2. The robustness of the results to changes in those environmental coefficients is explored in the sensitivity analysis.

### Scenarios

For each socio-demographic group, the model produces solution diets for increasingly stringent GHGE constraints. The first ‘Nutrition only’ scenario only imposes the nutritional constraints, thus ensuring nutritional adequacy of the diet without restricting GHGE. The second ‘GHGE –33 %’ and third ‘GHGE –50 %’ scenarios impose a reduction in GHGE of one-third and one-half, as compared with current levels, in addition to the nutritional constraints. Current diets are referred to as ‘FinDiet 2017’ in the tables and figures.

### Sensitivity analysis

A sensitivity analysis investigates the robustness of the simulated more climate-friendly dietary changes to three key assumptions of the model. First, the sensitivity of the simulated more climate-friendly diets to changes in the food-specific GHGE coefficients was evaluated. In our baseline model, a set of LCA-based GHGE coefficients that exclude land-use carbon dioxide (CO_2_) emissions was used. This is generally the practice in the current LCA studies and guidelines. However, in Finland, emissions from agricultural land contribute by nearly 50 % to the total GHGE of the Finnish food system^(26)^. Subsequently, another set of food-specific, life-cycle GHGE coefficients derived from the environmentally extended input–output model of the Finnish economy ENVIMAT^(27)^ was introduced. These data include GHGE from land-use sectors as reported in the national greenhouse gas inventory. While this inclusion significantly increases the GHGE coefficients of the domestic agricultural commodities and food products derived thereof, it does not affect GHGE coefficients for products like wild berries, fish and game. We point out that the purpose of this analysis is not to compare the two sets of GHGE coefficients but to assess how sensitive the simulations of diets are to a change in such coefficients.

Second, we investigate how relaxing the constraint on the beef-to-dairy ratio influences the results. While the initial constraint reflects the current reality, a lower beef-to-dairy ratio is allowed to challenge our implicit assumption of a perfectly inelastic excess demand for beef from Finland.

Finally, the sensitivity analysis considers the influence of the level of the Fe intake reference value on the results by raising it from its observed level in current diets (10 mg/capita per day for females) to the level specified in the Nordic Nutrition Recommendations for pre-menopausal women (15 mg/capita per day^(12)^; henceforth, quantities per capita will be abbreviated to ‘cap’ when specifying units of measurement).

## Results

The food composition of baseline and simulated diets are reported in tabular form for each sex, socio-demographic group and scenario in Appendix C. Appendix D presents the nutritional properties and GHGE of those diets.

### Nutritionally adequate diets and their greenhouse gas emissions

We first identified the main nutritional problems of current diets in Finland by comparing average nutrient intakes (Appendix D, Table D.1) to the recommended or safe daily intakes of macronutrients, vitamins and minerals imposed by the model (Appendix A, Table A.1). On that basis, we found that for both sexes, the average intake of fibre was insufficient and that the problem was quantitatively more significant for males (22 g/cap per day intake *v*. 35 g/cap per day recommendation) than females (20 g/cap per day *v*. 25 g/cap per day). Too much of dietary energy also originated from SFA (15 E% for men, 14 E% for females, *v*. 10 E% maximum recommendation) and too little from carbohydrates (39 E% for men, 41 E% for females, *v*. 45 E% minimum recommendation). Finally, for both sexes, there were excessive intakes of Na, although only marginally so for females (2·5 g/cap per day *v*. 2·4 g/cap per day recommendation)^(12)^, and insufficient folate intakes.

Next, we investigated potential synergies or trade-offs between nutritional adequacy and GHGE of the Finnish diet by comparing the GHGE of the ‘Nutrition only’ diets, which corrected the nutritional problems outlined above, with the GHGE of current diets for various sub-populations. Table 1 reports the results for an average adult. We found large synergies between improvements in nutritional adequacy of the diets and reductions in GHGE, which were robust across socio-demographic groups. Hence, the imposition of nutritional recommendations alone on an average Finnish male resulted in a drop from 5·3 kg/cap per day of CO_2_ equivalent (CO_2_e) to 3·9 kg /cap per day, or a 27 % decrease in GHGE. The diet of an average female contains less energy and produces less GHGE (3·8 kg/cap per day of CO_2_e) to start with, but the imposition of the nutritional recommendations also brought climate benefits, with a 15 % reduction in dietary GHGE. When considering sub-population groups, the reductions in GHGE for the ‘Nutrition only’ scenario varied very little across income quintiles. The results for educational groups were more heterogeneous but did not reveal any clear, monotonic relationship between educational level and GHGE reduction.

**Table 1:** GHGE of the current average diet and simulated nutritionally adequate diet of an average Finnish adult

Sex	Group	FinDiet 2017 (kg CO_2_e/cap per day)	Nutrition only (kg CO_2_e/cap per day)	Percentage difference
Male	All	5·30	3·87	−27·0 %
Male	Educ1	5·22	3·84	−26·4 %
Male	Educ2	5·50	3·69	−33·0 %
Male	Educ3	5·18	4·08	−21·3 %
Male	IncQ1	5·34	4·01	−24·9 %
Male	IncQ3	5·02	3·82	−24·0 %
Male	IncQ5	5·66	4·19	−26·0 %
Female	All	3·78	3·20	−15·5 %
Female	Educ1	3·63	3·05	−16·0 %
Female	Educ2	3·86	3·22	−16·7 %
Female	Educ3	3·86	3·32	−13·8 %
Female	IncQ1	3·49	2·91	−16·6 %
Female	IncQ3	3·70	3·23	−12·6 %
Female	IncQ5	4·01	3·37	−16·0 %

GHGE, greenhouse gas emissions.

Educ1–3 denote increasing educational categories. IncQ1–5 denote increasing income quintiles.

### Dietary adjustments of an average adult for nutritional adequacy and reduced greenhouse gas emissions

**Fig. 1:**
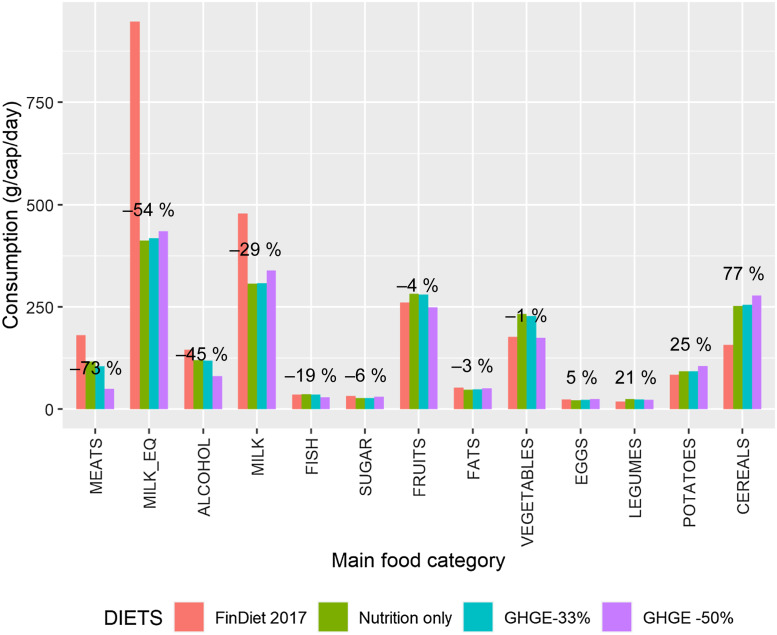
Changes in diets, average adult male. The figure next to each group of four bars gives the percentage change in consumption between the current situation as described by the FinDiet 2017 data and the optimised diet imposing all nutritional recommendations and a 50 % reduction in GHGE (i.e. scenario ‘GHGE –50 %’). The main food categories are described in terms of the seventy-four food categories in Table A.2. MILK_EQ is an aggregate of the food categories included in the MILK main food category, which uses milk equivalent coefficients for the aggregation. GHGE, greenhouse gas emissions

**Fig. 2:**
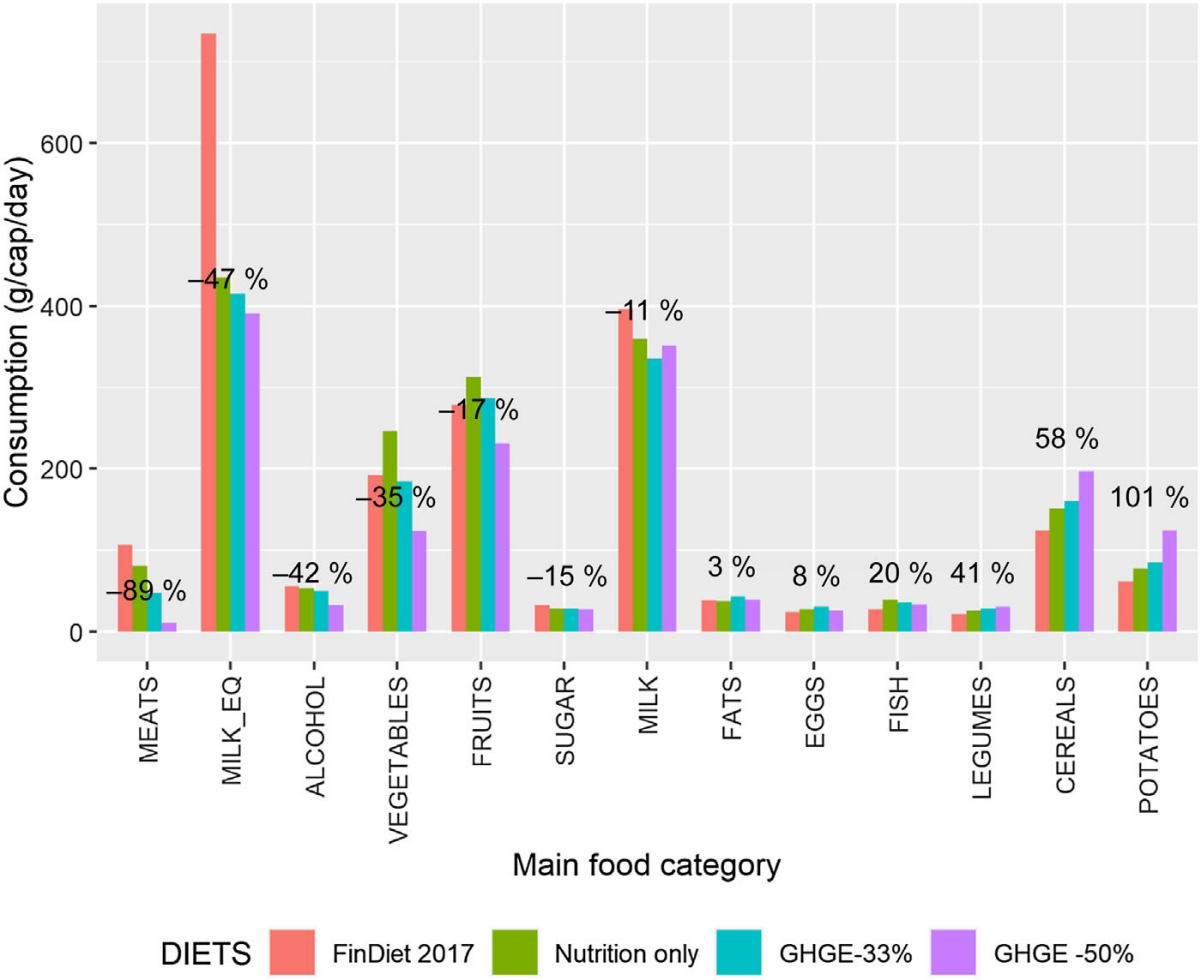
Changes in diets, average adult female. The figure next to each group of four bars gives the percentage change in consumption between the current situation as described by the FinDiet 2017 data and the optimised diet imposing nutritional recommendations and a 50 % reduction in GHGE (i.e. scenario ‘GHGE –50 %’). The main food categories are described in terms of the seventy-four food categories in Table A.2. MILK_EQ is an aggregate of the food categories included in the MILK main food category, which uses milk equivalent coefficients for the aggregation. GHGE, greenhouse gas emissions

The simulated diets for an average adult male and female across the seventy-four food categories are reported in Table C.1, but interpretation requires further aggregation of the food categories. Figures 1 and 2 present the results for thirteen main food categories and for an average male and female, respectively, with bars that compare the composition of the baseline diet (i.e. the FinDiet 2017 diet) and the three simulated scenarios. The figures show that, for most foods, the main adjustment was made to comply with the nutritional recommendations (green bars). Since the ‘Nutrition only’ scenario had already brought about a large reduction in GHGE, a few additional adjustments were necessary to achieve the 33 % reduction in GHGE (blue bars). Further tightening of the GHGE constraint (purple bars) then brought about some notable changes in the meat, cereals and potato categories. The primary mechanism for reducing the GHGE of the male diet was the substitution of meat (–73 %) and dairy products (–29 %), especially ripened cheese, with cereal products (+77 %) and potatoes (+25 %) and part of the vegetables, for example, roots (+54 %). The picture for an average female was qualitatively similar but quantitatively more extreme, with minimal consumption of meat (11 g/cap per day) under the strictest GHGE reduction scenario, and the calories from meat being replaced primarily by calories from cereals (+70 g/cap per day) but also potatoes (+63 g/cap per day) and roots (+52 %).

While the broad direction of substitutions among foods was in line with expectations based on previous research, the simulations also generated a nuanced picture of the dietary adjustments necessary to reduce GHGE while ensuring nutritional adequacy. First, with respect to the much-discussed issue of proteins, we note in Fig. 1 that the increase in consumption of protein-rich legumes was limited in both relative terms (+21 %) and absolute terms (4 g/cap per day) and that the ‘GHGE –50 %’ diet contained reduced quantities of fish (–19 %). The results for an average female (Fig. 2) only differ marginally, with fish consumption increasing moderately (+20 %) for the ‘GHGE –50 %’ scenario.

Turning to the dairy category, the substantial reduction in consumption was driven by the nutritional recommendations rather than the GHGE reductions of the simulated diets. Indeed, Fig. 1 shows a small *increase* in consumption of dairy products for the second and third scenarios compared with the baseline level in the data, but the increase occurs after a large decrease for the first scenario (–29 %, or –54 % in terms of milk equivalents). The absolute quantities of dairy products remain high (> 300 g/cap per day) in all diets. Inside the dairy products category, there can be seen a clear decrease, especially in ripened cheeses (Table C.1), which is reflected in the decrease in raw milk (milk equivalents).

The quantities of fruits and vegetables in the simulated diets corresponding to the three scenarios were very similar to those in the current diet (–4 and –1 %, respectively, for the ‘GHGE –50 %’ scenario in Fig. 1). This may reflect in part the fact that consumption of those food categories was already substantial among Finnish males on average (261 g/d per cap for fruits and 177 g/d per cap for vegetables).

**Fig. 3:**
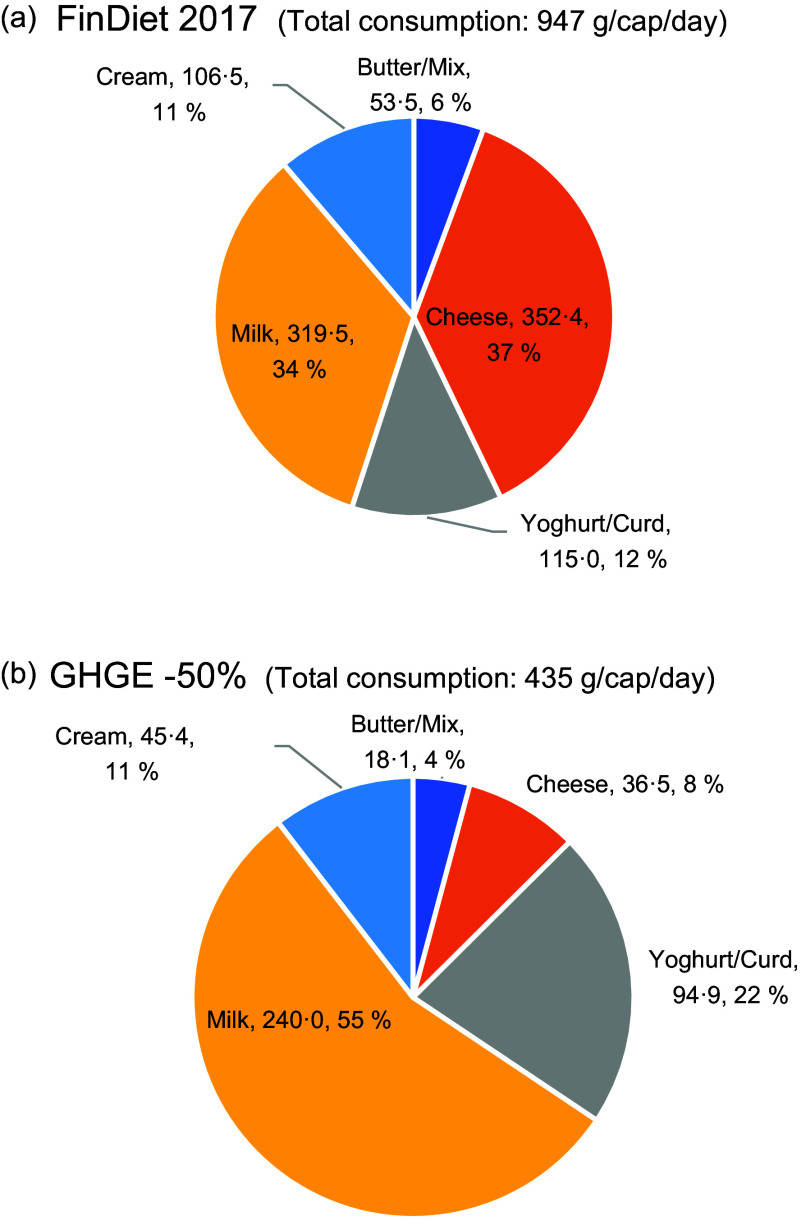
(a) (upper part) and (b) (lower part): Intra-category composition of dairy consumed by an average Finnish male in the current diet (upper part) and –50 % GHGE scenario (lower part) (absolute quantities in g/cap per day, expressed in milk equivalents). GHGE, greenhouse gas emissions

**Fig. 4:**
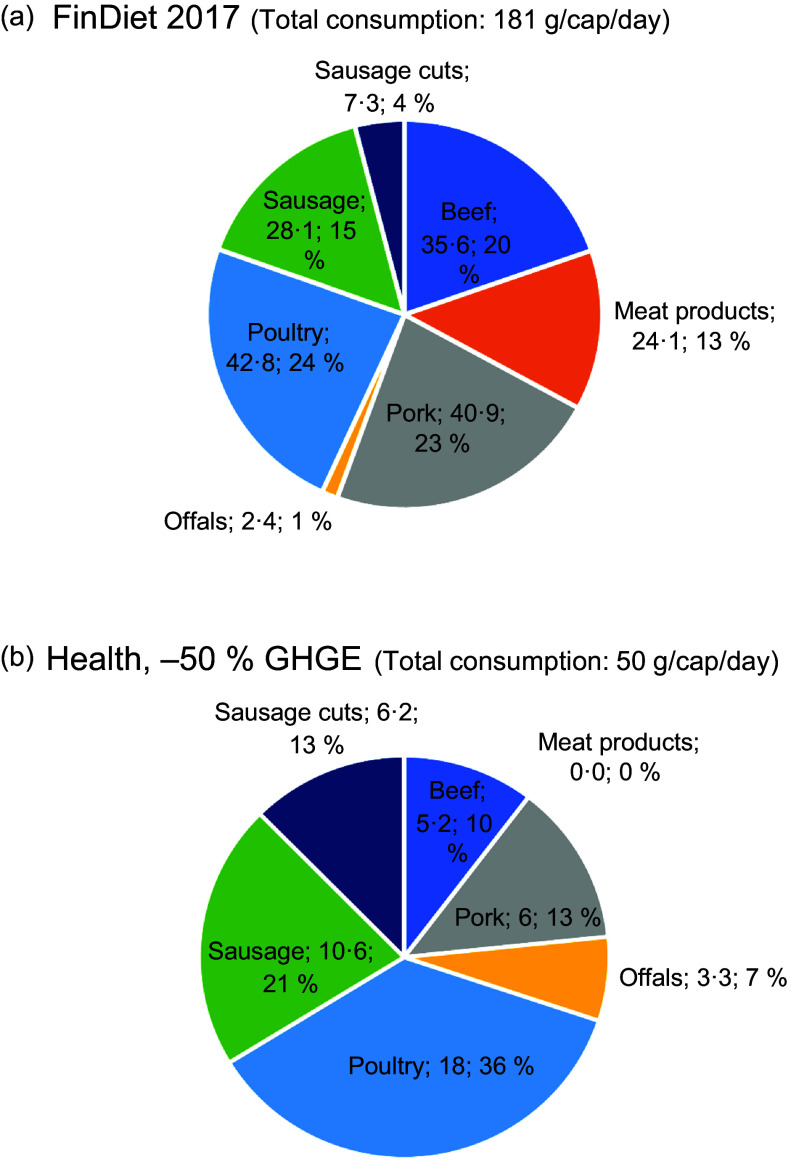
(a) (upper part) and (b) (lower part): Intra-category composition of meat consumed by an average Finnish male in the current diet (upper part) and –50 % GHGE scenario (lower part) (absolute quantities in g/cap per day). GHGE, greenhouse gas emissions

In addition to the changes in terms of broad categories outlined above, the secondary mechanism of dietary adjustment for GHGE reductions was the intra-category substitution of foods for one another. For instance, within the dairy category, the relative importance of liquid milk and yoghurt was much larger in the lower GHGE than in current diets (Fig. 3(a) and (b)), while the relative importance of ripened cheese decreased considerably as GHGE were reduced. The results for the meat category reported graphically in Fig. 4(a) and (b) and in full in Appendix C indicated a shift away from the consumption of beef and lamb towards poultry, offals and sausages, which is readily explained by the much higher GHGE of the foods originating from ruminants. At the sub-group level of vegetables, there was also an increase in root vegetables and decrease in fruiting vegetables (e.g. tomatoes typically grown in green houses) (Table C.1).

### Differences in dietary adjustments across socio-demographic groups

**Fig. 5:**
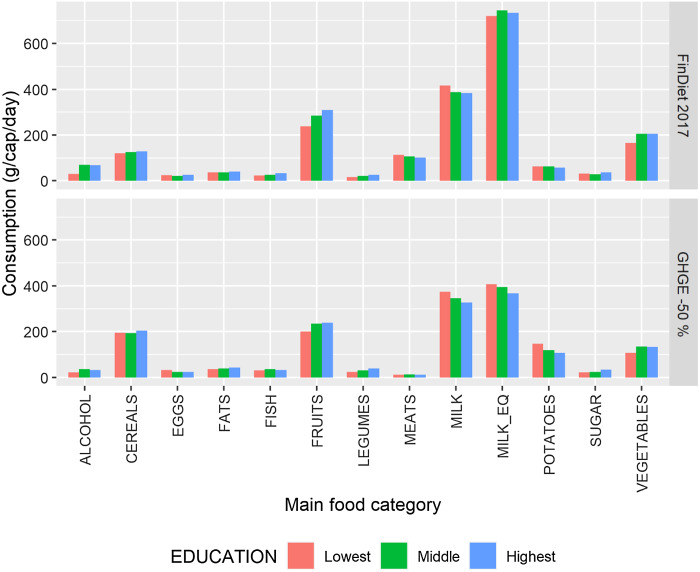
Differences in diets across educational levels, average Finnish female. The upper part of the graph presents the baseline diets and the lower part the simulated nutritionally adequate diet with a 50 % lower GHGE impact than the current diets. The main food categories are described in terms of the seventy-four food categories in Table A.2. MILK_EQ is an aggregate of the food categories included in the MILK main food category, which uses milk equivalent coefficients for the aggregation. GHGE, greenhouse gas emissions

We then analysed differences in initial diets and adjustments to more sustainable diets across socio-demographic groups, starting with educational categories. Figure 5 compares the diets of an adult female across the three educational categories at the baseline (upper section) and under the strictest GHGE reduction scenario (lower section). We first note an initial socio-economic gradient in the consumption of some foods, but that the gradient is not very large. Females in the highest category consumed substantially more fish (+41 %), legumes (+56 %), fruits (+29 %) and vegetables (+25 %) but also more alcohol (+131 %) compared with females in the lowest educational category. Those differences in diet composition were not particularly significant as far as GHGE are concerned.

The dietary adjustments for reduced GHGE (lower part of Fig. 5) followed the broad pattern described in section 3·2 for an average female: Considerable reductions in meat consumption were largely compensated, in terms of energy, by increases in consumption of cereals and potatoes. There were, however, some important nuances. A 50 % reduction in GHGE entailed a much larger increase in the consumption of potatoes for females in the lowest educational category (+134 % or 85 g/cap per day) than for females in the highest educational category (+85 % or 49 g/cap per day). Differences in dietary adjustments were also noticeable for some other food categories: eggs (+27 % for the lowest *v*. –8 % for the highest category), alcohol (–23 % *v*. –52 %), fish (+14 % *v*. –1 %) and sugar (–24 % *v*. –6 %). However, while some of those adjustments may appear substantial, the lower panel of Fig. 5 shows that the most climate-friendly diets remained very similar across educational groups.

**Fig. 6:**
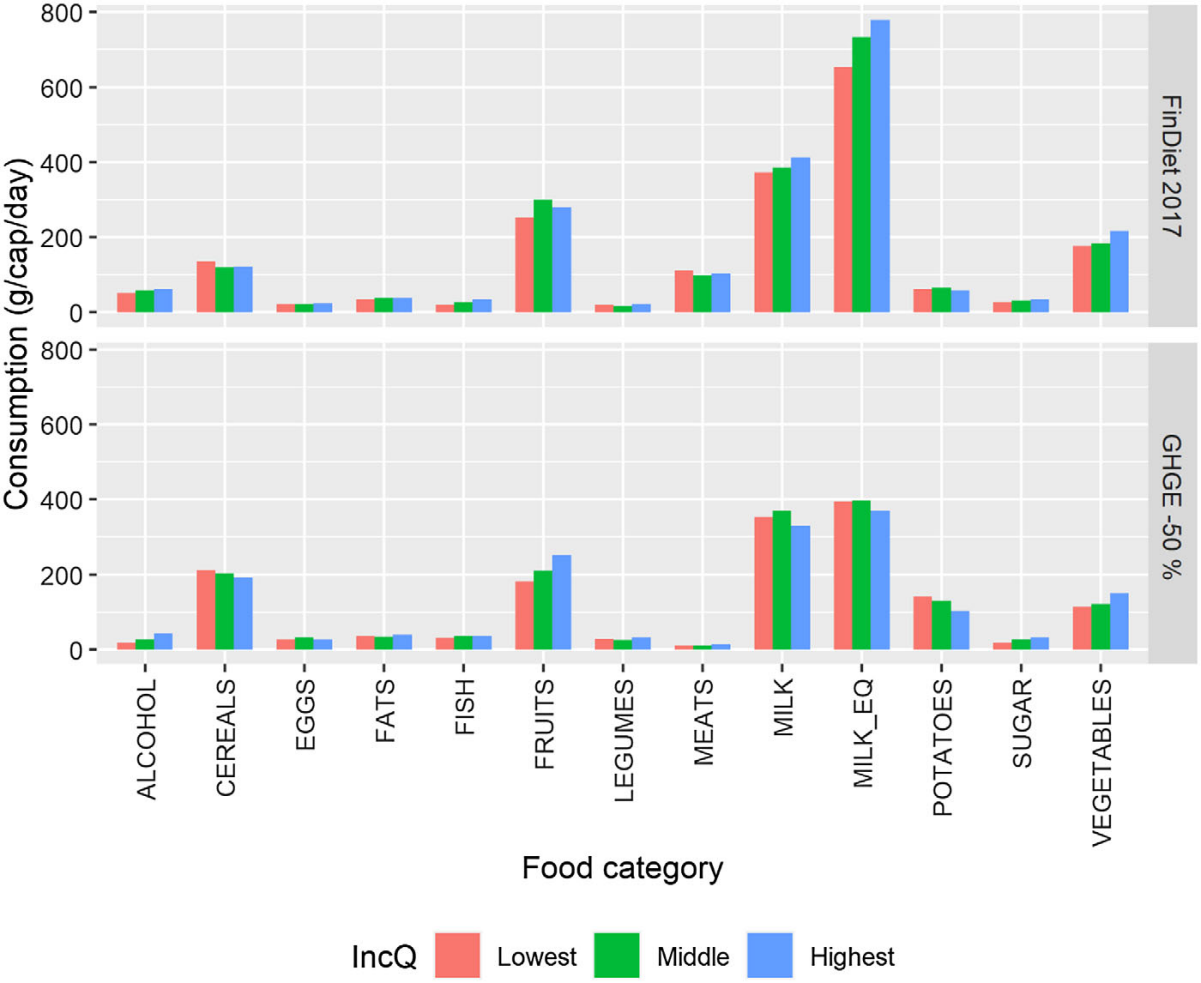
Differences in diets across income quintiles, average Finnish female. The upper part of the graph presents the baseline diets and the lower part the simulated nutritionally adequate diet with a 50 % lower GHGE impact than the current diets. The main food categories are described in terms of the seventy-four food categories in Table A.2. MILK_EQ is an aggregate of the food categories included in the MILK main food category, which uses milk equivalent coefficients for the aggregation. GHGE, greenhouse gas emissions

At this level of food aggregation, the simulated more climate-friendly diets for an average female also remained by and large very similar across income categories (Fig. 6). Under the ‘GHGE –50 %’ scenario, a positive income gradient in the consumption of fruits and a negative one in the consumption of potatoes appeared, but the magnitudes were not large. The other gradients in consumption observed in the current diet – for instance, for dairy products – disappeared in the lower-impact diet.

### Sensitivity analysis

Table 2 presents the sensitivity of the simulated GHGE to some of the key assumptions outlined in the methodology section. The inclusion of GHGE from agricultural land resulted in a larger total GHGE from current diets (+22 % for an average male and +31 % for an average female), but the two simulated ‘GHGE –50 %’ diets remained very similar, although we note some differences for the alcohol, meat and fruit categories. This is in line with the fact that the inclusion of GHGE from agricultural land increases the coefficients for both plant- and animal-based products derived from Finnish agriculture.

**Table 2:** Sensitivity analysis

	Average Male				Average Female				
	FinDiet 2017	GHGE –50 %			FinDiet 2017	GHGE –50 %			
Main food categories		LCA coeffs	IO coefficients	No beef/milk constraint		LCA coeffs	IO coefficients	No beef/milk constraint	Fe >= 15 mg
Alcohol	146	81	137	98	56	33	56	45	23
Cereals*	158	278	278	273	125	197	183	178	222
Eggs	24	25	27	24	24	26	32	30	65
Fats†	53	51	51	52	38	39	41	44	14
Fish	36	29	30	31	28	33	32	33	50
Fruits	261	249	284	260	279	232	312	271	310
Legumes‡	19	23	23	23	22	31	30	30	58
Meats	181	50	40	54	107	11	17	7	8
Milk§	478	339	320	331	395	351	302	353	275
Potatoes	85	106	100	100	62	124	92	108	134
Sugar	32	30	31	30	32	27	33	28	17
Vegetables	177	175	191	194	192	124	149	132	152
Milk_eq||	947	435	395	451	734	391	370	443	313

GHGE, greenhouse gas emissions.

The main food categories (meat, etc.) are described in terms of the seventy-four food ingredients in Table A.2.

* Includes all cereal products.

† Includes oils.

‡ Includes legumes, seeds and nuts.

§ Includes all dairy products in terms of physical quantity.

|| Includes all dairy products in terms of milk equivalents (i.e. uses milk equivalent coefficients for the aggregation).

Next we assessed the importance of the beef to dairy ratio constraint introduced into the model to capture the fact that beef production in Finland is largely a by-product of the dairy industry. A comparison of the ‘GHGE –50 %’ diets with and without that constraint in Table 2 indicated that the results did not depend strongly on that assumption.

Finally, we turned to the implications of raising the level of the habitual Fe intake for pre-menopausal females from 10 mg/cap per day to the recommended intake of 15 mg/cap per day. Additional simulations (not reported) indicated that under the ‘Nutrition only’ scenario, the GHGE *increased* as compared with the baseline when the higher level was imposed – that is, the synergy nutritional adequacy-climate disappeared due to this single constraint, which pushed consumption towards Fe-rich meat and towards fish, eggs and vegetables, all foods that have relatively high GHGE per calorie. Reconciling nutritional adequacy and low GHGE of the diet then became more difficult with the higher constraint level, and Table 2 shows that, accordingly, the ‘GHGE –50 %’ diet with the higher intake threshold has a different composition than the equivalent diet simulated with the lower intake threshold. Tightening the minimum level of Fe intake induced additional increases in consumption of eggs (65 g/cap per day *v*. 25 g/cap per day), fish (50 g/cap per day *v*. 32 g/cap per day), legumes (58 g/cap per day *v*. 31 g/cap per day), fruits, vegetables and cereals but further decreases in consumption of dairy products, meat, fat and sugar.

## Discussion and conclusions

Our analysis contributes to the ongoing debate on how much demand-side measures could realistically contribute to the decline in GHGE from the food system without compromising the nutritional adequacy of diets. We have established four key results in a Finnish context:

1. From the currently observed situation, there are win-win dietary changes that reduce GHGE and increase compliance with nutritional recommendations.
2. Significant reductions in GHGE can be achieved by adopting flexitarian diets that do not require the exclusion of entire food categories from consumption.
3. The main dietary changes involve the substitution of meat with cereals and potatoes and the intra-category substitution of foods, particularly beef with poultry in the meat category or cheese with yoghurt and milk in the dairy category.
4. Altogether, a one-third reduction in dietary GHGE represents a reasonable target for the transition to a climate-friendly Finnish food system, keeping in mind that considerable gains can also be achieved through changes in land use^(28)^ and technology^(29)^.

The most salient dietary changes, both across main food categories and within main food categories, are summarised in Table 3. Due to the limited space, the intra-category substitutions are only described for males in the table, but they are very similar for females.

**Table 3:** Summary of the main dietary adjustments, Δ*x*, to achieve a 33 % reduction in GHGE while complying with all nutritional constraints. All quantities consumed, denoted *x*, are in g/cap per day

Male	*x*	Δ*x*		Female	*x*	Δ*x*
Main food category	Initial diet	GHGE –33 %	Important intra-category substitutions	Main food category	Initial diet	GHGE –33 %
Milk	478	−170	Cheese with yoghurt and milk; High-fat cheese with low-fat cheese	Milk	395	−60
Meats	181	−76	Beef and lamb with poultry, offals, sausages	Meats	107	−59
Alcohol	146	−28		Vegetables	192	−8
Sugar	32	−5		Alcohol	56	−5
Fats	53	−4	Butter with oil, margarine	Sugar	32	−4
Eggs	24	−2		Fats	38	5
Fish	36	0		Eggs	24	7
Legumes	19	5		Legumes	22	7
Potatoes	85	9		Fruits	279	7
Fruits	261	19		Fish	28	8
Vegetables	177	51		Potatoes	62	23
Cereals	158	98	Rice with wheat, oats, rye	Cereals	125	35

GHGE, greenhouse gas emissions.

Although the synergies nutrition climate may have been expected, we note that the literature reports various counterexamples^(19,30–32)^ so that their presence and magnitude in a Finnish context could not be assumed *a priori.* The importance of the cultural and national context for the characterisation of sustainable diets is in line with the conclusion of MacDiarmid’s review of the literature^(33)^ on the subject or of a recent Swedish study^(34)^. Our study also fills a gap in the existing literature by showing that those synergies are present across the socio-demographic groups, regardless of sex, education or income, which will facilitate the formulation of clear win-win sustainable diet policies.

The assessment of whether policy targets are reasonable or not necessarily involves an element of judgement and subjectivity, but our conclusion draws primarily on two findings. Although lowering GHGE would require a broad reallocation of the diet from animal to plant-based products, the simulated ‘GHGE –33 %’ diets still contain large quantities of meat and dairy products (e.g. >100 g/cap per day of meat and >300 g/cap per day of dairy products for an average male) and therefore fall in the flexitarian category, at least according to some definitions (see Dagevos (2021)^(35)^ for a discussion). Tightening the GHGE reduction from 33 to 50 % would require considerable additional reductions in meat consumption, in particular for females (an almost 90 % reduction from the baseline), which probably make those population-level dietary adjustments unrealistic, at least in the short to medium term. Those results and their interpretation for policy action are consistent with those derived in a French context by Perignon *et al.* (2016)^(36)^.

We acknowledge that our study does not allow for a full investigation of the equity impacts of dietary changes, not least because we have not analysed diet costs explicitly due to the lack of price information compatible with the food categorisation in the optimisation model. We note, however, that the broad direction of substitutions, both across categories (e.g. cereals and potatoes for meat) and within categories (milk for cheese, poultry for beef) implies that more climate-friendly diets are unlikely to be costlier than current ones. This is reassuring given that many studies in public health nutrition have identified diet cost as a major barrier to dietary change^(37)^. It is also in line with the conclusion of a recent study of German diets that found that health-promoting, culturally acceptable diets with lower GHGE, derived through linear programming, cost less than the baseline German diet^(38)^.

In addition to those overarching conclusions, the study generates a number of new and specific insights on sustainable diets in a Finnish context. Although much of the public and policy debate about dietary change focuses on proteins, we find that none of the constraints on the amino acid composition and quantity of protein is binding in the simulated diets. Further, it is worth noting that the food-habit constraints for the food categories containing pulses/legumes are not binding either (Appendix C), so the result of a relatively small increase in pulse and legume consumption is not driven by those constraints. Altogether, the results imply that protein intakes are not an issue when seeking to reconcile nutritional adequacy and GHGE of diets. Thus, the loss of proteins caused by the decrease in consumption of animal products does not create major nutritional problems, neither in terms of protein quantity nor composition. We explain this result by the following: (i) The large levels of intakes of proteins in initial diets so that significant reductions in intakes are compatible with minimum recommended intakes. Indeed, the detailed results for males show that the ‘GHGE –50 %’ scenario produces nutritionally adequate diets containing 20 % less proteins than current diets, which remains above minimum recommended intakes; and (ii) The fact that cereal products are themselves rich in proteins and their efficiency in terms of protein made available for human consumption per unit of climate impact has been demonstrated previously^(39)^. Thus, it seems that the misconceptions regarding the role of protein in sustainable diets already pointed out by MacDiarmid^(33)^, such as the overestimation of the protein requirements for a healthy diet, remain prevalent and should be addressed more directly by scientists. There may be, though, vulnerable population groups, for example, the elderly above the age of 65 years, whose protein needs are increased^(12,13)^, and more research is needed to evaluate the protein adequacy of GHGE-reduced diets in these age groups. Further disaggregation of the cereal food categories would also make it possible to investigate the relative importance of whole-grain cereal products in nutritionally adequate and climate-friendly diets.

According to the results of the simulations, the substitutions necessary to achieve better nutritional adequacy and lower GHGE are more subtle than just ‘more plants, less animals’. Hence, halving the GHGE of diets requires considerable reductions in meat consumption, but it is also compatible with moderate levels of consumption of dairy products. On the plant side, the model suggests that increasing consumption of fruits and vegetables is not a key priority to achieve the 50 % reduction in GHGE while keeping diets nutritionally adequate. This point has been made previously in several studies of sustainable diets, with, for instance, Vieux *et al.* (2012)^(31)^ concluding their analysis of self-selected diets in France by stating that ‘substituting fruit and vegetables for meat (especially deli meat) may be desirable for health but is not necessarily the best approach to decreasing diet-associated greenhouse gas emissions’. Irz and Kurppa (2013)^(30)^ concluded along similar lines in their analysis of Finnish food consumption. In line with Tuomisto (2019)^(40)^, we therefore urge analysts, policymakers and other stakeholders of the food system to integrate the complexity of sustainable diets when making decisions.

Finally, our analysis presents some limitations that open the door to future research. Although our model features some nutritional, climate and social dimensions, the analysis remains perfectible, and other elements would ideally be captured. First, regarding its coverage, the analysis was limited to the adult population. Extending it to other age groups would be useful for gaining an overall picture and supporting national climate policy, for example. Further, in some cases, a finer breakdown of the adult population considered in the analysis would also be necessary. Hence, a critical nutrient that is challenging to consider in an optimisation framework is Fe due to the very different dietary requirements of sub-population groups, for example, men and pre- and post-menopausal females. Even among pre-menopausal females, who have the highest Fe requirements, variation is large, for example, due to different degrees of menstrual blood losses or the use of contraceptives, which result in a decrease in blood losses^(12)^. In this study, we ended up using as the minimum Fe requirement among all females 10 mg/d, which is the average intake of all females in the latest National Dietary Survey of Finland^(41)^. This is sufficient for post-menopausal females and the average requirement reference value (average requirement, median of the assumed requirement distribution) of Fe intake for pre-menopausal females^(12)^ but insufficient for part (50 %) of the pre-menopausal females to cover Fe losses in the population group. Thus, a limitation of this study may be that the results are not fully applicable to pre-menopausal females. Our sensitivity analysis shows that reconciling nutritional adequacy and low GHGE becomes much more difficult when Fe requirements are increased to Nordic Nutrition Recommendations levels, which raises the broader question of the role of nutritional supplements in sustainable diets, which to date has not received enough attention.

There are many other directions to extend and improve the analysis. In the environmental domain, we know that food systems contribute significantly to the breach of many planetary boundaries, in particular linked to biodiversity and quantity and quality of water resources^(1)^. Adding other environmental constraints to the optimisation model is technically possible, but the practical difficulty lies with the lack of food-specific environmental impact coefficients applicable to the Finnish context. On the economic side, the explicit consideration of diet costs, which requires the matching of food classifications across databases (e.g. dietary intake survey *v*. household budget survey), should be a priority to allow further analysis of diet affordability and equity impacts. Finally, it must be acknowledged that the issue of cultural acceptability and potential for adoption of the simulated diets are only partially addressed in our model. The development of an objective function that better captures the difficulty for consumers of substituting foods for one another, as proposed by Green *et al.* (2015)^(42)^, appears promising to improve the model. Regardless of the improvements in the quantitative methods used to characterise sustainable diets, there is also a need for qualitative work with consumers and ordinary citizens in order to understand the real potential for and obstacles to the adoption of those diets.

## Supporting information

Irz et al. supplementary material Irz et al. supplementary material

## Footnotes

* See Finnish Institute for Health and Welfare. National Food Composition Database FINELI®, Release 20. Open-access version available online: https://fineli.fi/fineli/en/index?

## Supplementary material

For supplementary material accompanying this paper visit https://doi.org/10.1017/S1368980024000508.

## References

[1] Campbell BM , Beare DJ , Bennett EM et al. (2017) Agriculture production as a major driver of the earth system exceeding planetary boundaries. Ecol Soc 22, 8.

[2] Food and Agriculture Organization of the United Nations, World Health Organisation (2019) Sustainable Healthy Diets – Guiding Principles. Rome, Italy: FAO.

[3] Willett W , Rockström J , Loken B et al. (2019) Food in the Anthropocene: the EAT–Lancet Commission on healthy diets from sustainable food systems. Lancet 393, 447–492. 30660336 10.1016/S0140-6736(18)31788-4

[4] von Braun J , Afsana K , Fresco LO et al. (2023) Science and Innovations for Food Systems Transformation. Cham: Springer Nature. 38285810

[5] European Commission (2020) Towards a Sustainable Food System. Brussels, Belgium: EC, Directorate-General for Research and Innovation.

[6] Carlsson-Kanyama A & González AD (2009) Potential contributions of food consumption patterns to climate change. Am J Clin Nutr 89, 1704S–1709S. 19339402 10.3945/ajcn.2009.26736AA

[7] Gazan R , Brouzes CMC , Vieux F et al. (2018) Mathematical optimization to explore tomorrow’s sustainable diets: a narrative review. Adv Nutr 9, 602–616. 30239584 10.1093/advances/nmy049 PMC6140431

[8] Shepherd R (1999) Social determinants of food choice. Proc Nutr Soc 58, 807–812. 10817147 10.1017/s0029665199001093

[9] Yin J , Yang D , Zhang X et al. (2020) Diet shift: considering environment, health and food culture. Sci Total Environ 719, 137484. 32135323 10.1016/j.scitotenv.2020.137484

[10] Perignon M & Darmon N (2022) Advantages and limitations of the methodological approaches used to study dietary shifts towards improved nutrition and sustainability. Nutr Rev 80, 579–597. 35142357 10.1093/nutrit/nuab091 PMC8829675

[11] Vozoris N , Davis B & Tarasuk V (2002) The affordability of a nutritious diet for households on welfare in Toronto. Can J Public Heal 93, 36–40. 10.1007/BF03404415 PMC6979865 11925698

[12] Nordic Council of Ministers (2014) Nordic Nutrition Recommendations 2012: Integrating Nutrition and Physical Activity. Copenhagen, Denmark: Norden.

[13] National Nutrition Council (2014) Terveyttä ruoasta - Suomalaiset Ravitsemussuositukset 2014 (Finnish Nutrition Recommendations 2014). Tampere, Finland: Valtion ravitsemusneuvottelukunta.

[14] Srinivasan CS , Irz X & Shankar B (2006) An assessment of the potential consumption impacts of WHO dietary norms in OECD countries. Food Policy 31, 53–77.

[15] World Health Organisation (2007) Protein and Amino Acid Requirements in Human Nutrition: Report of a Joint WHO/FAO/UNU Expert Consultation. World Heal. Organ. Tech. Rep. Ser. ProQuest Ebook Central. https://ebookcentral.proquest.com/lib/helsinki-ebooks/detail.action?docID=305231 (accessed May 2023). 18330140

[16] Lahti-Koski M , Valsta LM , Alfthan G et al. (2003) Iron status of adults in the capital area of Finland. Eur J Nutr 42, 287–292. 14564462 10.1007/s00394-003-0425-3

[17] Fogelholm M , Alopaeus K , Silvennoinen T et al. (1993) Factors affecting iron status in non-pregnant women from urban South Finland. Eur J Clin Nutr 47, 567–574. 8404793

[18] Valsta LM , Tapanainen H , Kortetmäki T et al. (2022) Disparities in nutritional adequacy of diets between different socioeconomic groups of Finnish adults. Nutrients 14, 1347. 35405960 10.3390/nu14071347 PMC9002951

[19] Vieux F , Perignon M , Gazan R et al. (2018) Dietary changes needed to improve diet sustainability: are they similar across Europe?. Eur J Clin Nutr 72, 951–960. 29402959 10.1038/s41430-017-0080-z PMC6035144

[20] Lehtonen H & Irz X (2013) Impacts of reducing red meat consumption on agricultural production in Finland. Agric Food Sci 22, 356–370.

[21] Broekema R , Tyszler M , van’t Veer P et al. (2020) Future-proof and sustainable healthy diets based on current eating patterns in the Netherlands. Am J Clin Nutr 112, 1338–1347. 32766880 10.1093/ajcn/nqaa217 PMC7657328

[22] Turlach BA & Weingessel A (2019) Quadprog: Functions to Solve Quadratic Programming Problems. R package version 1.5–8. https://cran.r-project.org/web/packages/quadprog/quadprog.pdf (accessed August 2021).

[23] Kaartinen N , Tapanainen H , Reinivuo H et al. (2020) The Finnish national dietary survey in adults and elderly (FinDiet 2017). EFSA Support Publ 17, 1–26.

[24] Borodulin K , Sääksjärvi K (2019) *FinHealth 2017 study: methods* . In Finnish Inst Heal Welf. Helsinki, Finland: THL.

[25] Saarinen M , Kaljonen M , Niemi J et al. (2019) Ruokavaliomuutoksen vaikutukset ja muutosta tukevat politiikkayhdistelmät: RuokaMinimi-hankkeen loppuraportti. Helsinki, Finland: Valtioneuvoston kanslia.

[26] Kaljonen M , Karttunen K , Kortetmäki T (2022) *Oikeudenmukainen ruokamurros* . In Suom. ympäristö-keskuksen Rap.. Helsinki, Finland: SYKE.

[27] Seppälä J , Mäenpää I , Koskela S et al. (2011) An assessment of greenhouse gas emissions and material flows caused by the Finnish economy using the ENVIMAT model. J Clean Prod 19, 1833–1841.

[28] Lehtonen H , Huan-Niemi E & Niemi J (2022) The transition of agriculture to low carbon pathways with regional distributive impacts. Environ Innov Soc Transitions 44, 1–13.

[29] Parodi A , Leip A , De Boer IJM et al. (2018) The potential of future foods for sustainable and healthy diets. Nat Sustain 1, 782–789.

[30] Irz X , Kurppa S (2013) *Variations in environmental impact of food consumption in Finland. MTT Discuss.* Pap. 1–2013. Helsinki, Finland: MTT.

[31] Vieux F , Darmon N , Touazi D et al. (2012) Greenhouse gas emissions of self-selected individual diets in France: changing the diet structure or consuming less?. Ecol Econ 75, 91–101.

[32] Conrad Z , Drewnowski A , Belury MA et al. (2023) Greenhouse gas emissions, cost, and diet quality of specific diet patterns in the United States. Am J Clin Nutr 117, 1186–1194. 37075848 10.1016/j.ajcnut.2023.04.018

[33] MacDiarmid JI (2013) Is a healthy diet an environmentally sustainable diet?. Proc Nutr Soc 72, 13–20. 23186839 10.1017/S0029665112002893

[34] Eustachio Colombo P , Elinder LS , Lindroos AK et al. (2021) Designing nutritionally adequate and climate-friendly diets for omnivorous, pescatarian, vegetarian and vegan adolescents in Sweden using linear optimization. Nutrients 13, 2507. 34444667 10.3390/nu13082507 PMC8398609

[35] Dagevos H (2021) Finding flexitarians: current studies on meat eaters and meat reducers. Trends Food Sci Technol 114, 530–539.

[36] Perignon M , Masset G , Ferrari G et al. (2016) How low can dietary greenhouse gas emissions be reduced without impairing nutritional adequacy, affordability and acceptability of the diet? A modelling study to guide sustainable food choices. Public Health Nutr 19, 2662–2674. 27049598 10.1017/S1368980016000653 PMC10448381

[37] Drewnowski A & Eichelsdoerfer P (2009) Can low-income Americans afford a healthy diet?. Nutr Today 44, 246–249. 10.1097/NT.0b013e3181c29f79 PMC2847733 20368762

[38] Masino T , Colombo PE , Reis K et al. (2023) Climate-friendly, health-promoting, and culturally acceptable diets for German adult omnivores, pescatarians, vegetarians, and vegans – a linear programming approach. Nutr 109, 11197. 10.1016/j.nut.2023.111977 36801703

[39] González AD , Frostell B & Carlsson-Kanyama A (2011) Protein efficiency per unit energy and per unit greenhouse gas emissions: potential contribution of diet choices to climate change mitigation. Food Policy 36, 562–570.

[40] Tuomisto HL (2019) The complexity of sustainable diets. Nat Ecol Evol 3, 720–721. 30988495 10.1038/s41559-019-0875-5

[41] Kaartinen N , Tapanainen H , Männistö S et al. (2021) Aikuisväestön ruoankäytön ja ravintoaineiden saannin muutokset vuosina 1997–2017: kansallinen FinRavinto-tutkimus. Lääkärilehti 76, 273–280.

[42] Green R , Milner J , Dangour AD et al. The potential to reduce greenhouse gas emissions in the UK through healthy and realistic dietary change. Clim Change 129, 253–265.

