# Supplementary material for: Reducing the carbon footprint of diets across socio-demographic groups in Finland: a mathematical optimisation study
Source: Public Health Nutr. 2024 Mar 4;27(1):e98. doi: 10.1017/S1368980024000508 (PMC10993064; doi:10.1017/S1368980024000508)

**Appendix A – Parameters of the model**

**Table A.1** Nutrient recommendations imposed in the optimization. All quantities are expressed per person per day.

.
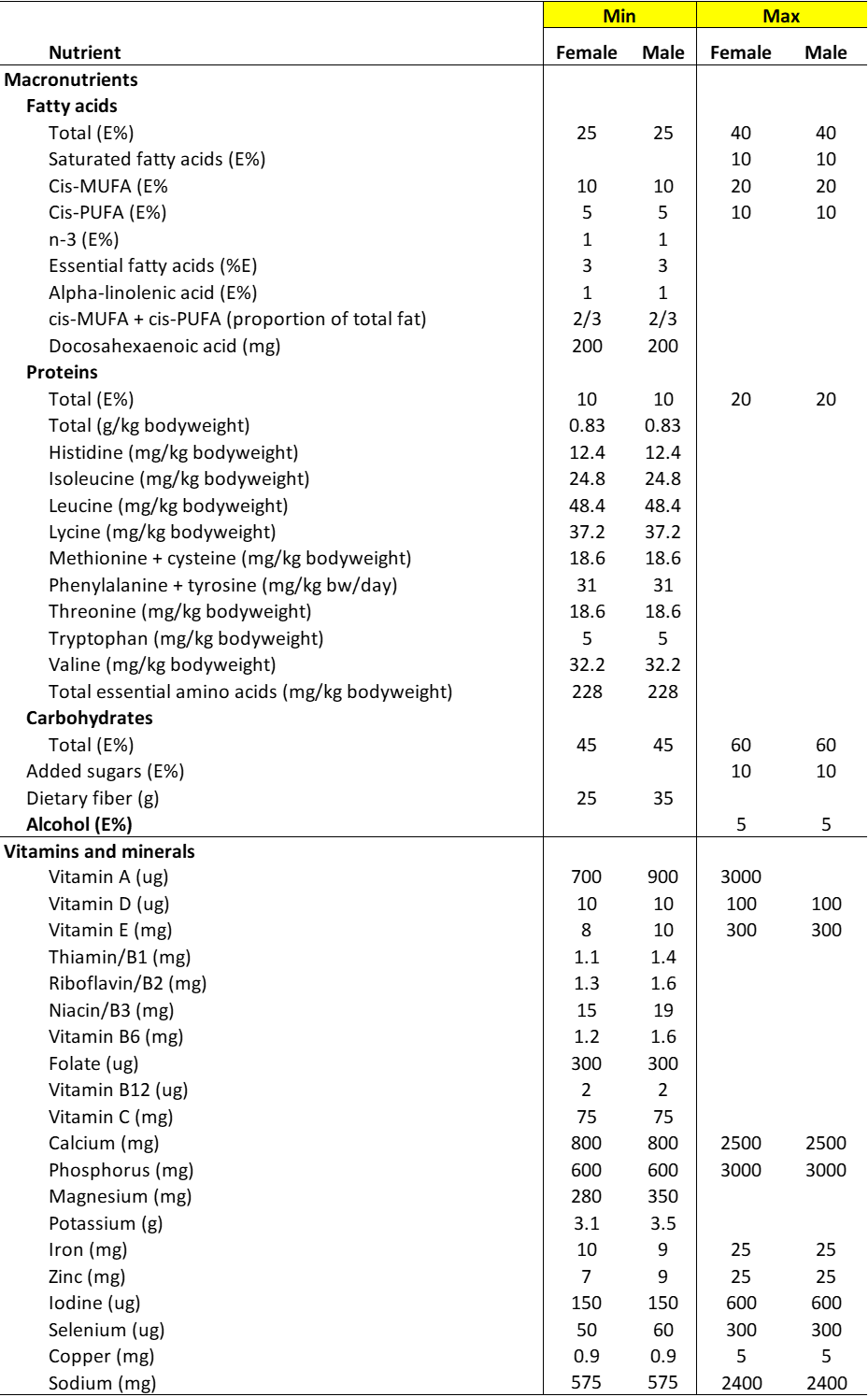


**Table A.2**: LCA coefficients (kg CO_2_e/kg food) and description of the 74 food categories

|  |  |  | **LCA Coefficients** | |
| --- | --- | --- | --- | --- |
| **Food category** | **Description** | **Main food category** | **Male** | **Female** |
| APPLE | Malaceous fruit | FRUIT | 0.41 | 0.41 |
| BARLEY | Barley and barley products | CER | 0.52 | 0.52 |
| BEEFLAMM | Beef and lamb/mutton | MEAT | 32.23 | 32.37 |
| BEERCIDER | Beer, cider and long drink | ALC | 1.35 | 1.20 |
| BERRY | Berries | FRUIT | 0.94 | 0.94 |
| BUTTER | Butter, milk fat | FAT | 19.08 | 19.08 |
| BUTTMIX | Blended spread | FAT | 7.30 | 7.29 |
| CABBAGE | Cabbages | VEG | 0.39 | 0.39 |
| CERLELIQ | Cereal/oat/soya/nut/seed drinks | CER | 0.66 | 0.67 |
| CEROTH | Other grains (incl. cereal bars) | CER | 2.21 | 2.25 |
| CHEECUPRHI | Cheese, ripened or processed > 17 % | MILK | 13.24 | 13.24 |
| CHEECUPRLO | Cheese, ripened or processed <=17 % | MILK | 13.18 | 13.20 |
| CHEEUNHI | Cheese, unripened, fresh cheese >15 % | MILK | 8.27 | 8.27 |
| CHEEUNLO | Cheese, unripened, fresh cheese <=15 % | MILK | 6.54 | 6.54 |
| CHOCOLAT | Chocolate | SUGAR | 4.40 | 4.40 |
| CITRUS | Citrus fruit | FRUIT | 0.30 | 0.30 |
| COFFEE | Coffee | BEV | 0.39 | 0.39 |
| CREAM | Cream | MILK | 3.07 | 3.07 |
| CURD | Quark | MILK | 1.22 | 1.22 |
| EGG | Chicken eggs | EGG | 2.81 | 2.81 |
| FATCOOK | Cooking and industrial fat/animal fat | FAT | 6.24 | 6.24 |
| FBDRINK | Juice drink | FRUIT | 1.13 | 1.13 |
| FISH | Fish, crustaceans and molluscs | FISH | 4.29 | 4.39 |
| FISHPROD | Fish products | FISH | 3.93 | 3.93 |
| FLAVSAUC | Condiments | FLAV | 0.77 | 0.77 |
| FLAVSEED | Dried spices and herbs | FLAV | 1.17 | 1.12 |
| FRUITCAN | Canned fruits | FRUIT | 1.51 | 1.51 |
| FRUITOTH | Other fruits | FRUIT | 1.15 | 1.15 |
| ICECREAM | Ice cream | MILK | 2.51 | 2.51 |
| INGRMIS | Miscellaneous ingredients | INGR | 0.00 | 0.00 |
| JAM | Jams | SUGAR | 1.60 | 1.60 |
| JUICE | Juices, incl. vegetable juices | FRUIT | 1.08 | 1.08 |
| MEATPROD | Cold cuts, meat products | MEAT | 16.72 | 16.72 |
| MILKFF | Milks skimmed | MILK | 0.94 | 0.94 |
| MILKHF | Milks >2% fat | MILK | 0.94 | 0.94 |
| MILKINGR | Milk powders | MILK | 15.20 | 15.20 |
| MILKLF | Milks <= 2% fat | MILK | 0.97 | 0.97 |
| MUSHRO | Edible fungi | VEG | 2.52 | 2.52 |
| NUTSEED | Nuts and seeds | LEGU | 1.31 | 1.31 |
| OAT | Oat and oat products | CER | 1.34 | 1.34 |
| OFFAL | Offal | MEAT | 15.18 | 15.18 |
| OIL | Oils | FAT | 3.82 | 3.82 |
| PEABEAN | Pulses vegetables, pulse products | LEGU | 1.84 | 1.85 |
| PORKGAME | Pork and game | MEAT | 6.38 | 6.36 |
| POTAPROD | Potato products | POTA | 0.49 | 0.49 |
| POTATO | Potato | POTA | 0.10 | 0.10 |
| POULTRY | Birds | MEAT | 5.49 | 5.49 |
| RICE | Rice | CER | 2.95 | 2.95 |
| ROOT | Root vegetables and tubers | VEG | 0.18 | 0.18 |
| RYE | Rye | CER | 1.08 | 1.08 |
| SALADDRE | Salad dressings and mayonnaises | FAT | 4.02 | 4.02 |
| SALT | Salt | INGR | 0.00 | 0.00 |
| SAUSAGE | Sausages | MEAT | 8.42 | 8.42 |
| SAUSCUTS | Cold cuts, sausages | MEAT | 8.42 | 8.42 |
| SOCUMILK | Soured and cultured milks | MILK | 1.22 | 1.22 |
| SOFTODRINK | Soft drinks and other drinks | BEV | 0.33 | 0.33 |
| SOUCREAM | Fermented milk products, other | MILK | 1.94 | 1.94 |
| SOYAPROD | Soya products | LEGU | 2.75 | 2.75 |
| SPECFOOD | Products for nutritional support, sport | DIET | 0.00 | 0.00 |
| STARCH | Starches | CER | 1.47 | 1.47 |
| SUGARSYR | Sugar and syrups | SUGAR | 3.13 | 3.13 |
| SWEAGE | Sweeteners | INGR | 0.00 | 0.00 |
| SWEET | Non-chocolate confectionery | SUGAR | 2.82 | 2.82 |
| TEA | Tea | BEV | 0.27 | 0.27 |
| VEGCANN | Canned vegetables | VEG | 1.80 | 1.80 |
| VEGFATHI | Margarine and fat spread >= 55% | FAT | 3.35 | 3.35 |
| VEGFATLO | Margarine and fat spread < 55% | FAT | 2.84 | 2.84 |
| VEGFRU | Fruit vegetables | VEG | 2.12 | 2.12 |
| VEGLEAF | Leaf vegetables | VEG | 2.20 | 2.20 |
| VEGONI | Onion-family vegetables | VEG | 0.31 | 0.31 |
| WATER | Water | BEV | 0.00 | 0.00 |
| WHEAT | Wheat | CER | 1.38 | 1.38 |
| WINESPIRIT | Wines and spirits | ALC | 1.28 | 1.22 |
| YOGHUR | Yoghurt | MILK | 1.59 | 1.59 |

**Appendix B:** Implementation of optimization model in R.

The model is implemented with R’s quadratic programming package quadprog, which allows for minimum constraints and equality constraints, as described in the package’s documentation (Berwin et al., 2019). Using the package requires setting up the problem in the following format:

$$\underset{b}{Min}(-d^{'}.b+\frac{1}{2b^{'}}.D.b)$$

subject to the constraints: $A^{'}.b\geq b_{0}$

where:

- *b* is the vector to be optimized
- *d* is a vector in the quadratic function to be minimized
- *D* is a matrix appearing in the quadratic function to be minimized
- *A* is a matrix defining the linear minimum or equality constraints
- *b_0_* are the levels of the constraints

The original diet optimization problem (1) is defined in terms of the consumption of food *x*. We redefine it in terms of the percentage changes in food consumption, that is, in terms of the variables $v_{i}=\frac{x_{i}-x_{i}^{0}}{x_{i}^{0}}$, which gives $x_{i}=x_{i}^{0}\left( 1+v_{i} \right)$. The **objective function** becomes $\underset{x}{Min}\sum_{i=1}^{n} v_{i}^{2}$, which is expressed in quadratic programming terms by setting:

- $b=v$ is a *Nx1* vector of percentage changes in consumption of each food *i* (with starting values set to zero).
- $d^{'}=\left( 0,\ldots, 0 \right)$ is a *Nx1* vector of zeros
- $D=\left( \begin{matrix} 2 & \cdots& 0 \\ \vdots& \ddots& \vdots\\ 0 & \cdots& 2 \end{matrix} \right)$ is a *NxN* matrix.

The equality constraint(s) (e.g., for energy) are introduced first in quadprog and the argument meq=q is given to the function to instruct R to treat those first *q* constraints as equalities. Mathematically, for instance for energy, equality means: $\sum_{i=1}^{n} a_{i}^{e}x_{i}^{0}v_{i}=0$. In this case the coefficients of the matrix *A* are simply the vector formed of terms $a_{i}^{e}x_{i}^{0}$ for each ingredient class, i.e. the energy derived from each ingredient class in the original diet.

The minimum constraints for absolute quantities of nutrients, for instance for vitamin A, are expressed in terms of the *v* variables: $\sum_{i=1}^{n} a_{i}^{j}x_{i}^{0}v_{i}\geq c^{j}-\sum_{i=1}^{n} a_{i}^{j}x_{i}^{0}$. Thus, for any nutrient *j* with a minimum constraint, the left hand side (LHS) of this equation gives the coefficients of matrix A for that constraint and correspond to the initial intakes of the constrained nutrient *j* from each food category *i*. The right hand side (RHS) of the equation are the gaps, or distance to the minimum permissible level of nutrient *j*. If not enough of nutrient *j* is consumed, that distance/gap is positive and measures the quantity deficit of nutrient *j*. Those gaps define the levels of the minimum constraints *b_0_* in quadprog.

Similarly, absolute maximum constraints, for nutrients or climate indicators, are first transformed into minimum constraints by inverting them (i.e., multiplying them by -1) and subsequently expressed as a function of our *v* variables: $\sum_{i=1}^{n} {-a}_{i}^{k}x_{i}^{0}v_{i}\geq-b^{j}+\sum_{i=1}^{n} a_{i}^{k}x_{i}^{0}$. For each *k* maximum constraint, the coefficients of matrix A are the negatives of the initial quantities of constrained nutrient *k* from each food *i*. The initial level of the constraint is the RHS term, which measures the negative of the distance to the maximum permissible level of nutrient *j*. Hence, positive values indicate that too much is already consumed.

Some nutrient requirements are expressed as a percentage of energy but, given that energy is held constant, it means that we also impose minimum or maximum quantities on those nutrients, and the previous equations are easily adjusted.

Non-negativity constraints on the food quantities are equivalent to imposing a -1 minimum constraint on the *v* variables. The constraint coefficients are therefore an *nxn* identity matrix, and the constraint levels are an n-vector of -1s.

Lower bound and upper-bound constraints on individual consumption are imposed by applying a similar logic.

It is worth noting that quadratic programming algorithms generate local optima that may miss superior solutions. However, the structure of the diet optimization problem as defined above is very specific and ensures that this is not a possibility here. More specifically, the matrix D defining the quadratic term of the objective function is simply two times the identity matrix, which is obviously symmetric positive definite. This makes the quadratic programming problem strictly convex, and such problems have a single global minimum, as established in general by Boyd (2009) and noticed elsewhere in the diet optimization literature (e.g., Persson et al. 2018).

Boyd S, Vandenberghe L. Convex optimization. 7th ed. Cambridge: Cambridge University Press; 2009.

Persson, M., Fagt, S., Pires, S. M., Poulsen, M., Vieux, F., & Nauta, M. J. (2018). Use of mathematical optimization models to derive healthy and safe fish intake. The Journal of nutrition, 148(2), 275-284.

**Appendix C – Detailed Baseline and Simulated Diets**

**Table C.1:** Initial and optimized diets (g/cap/day), average adult Finnish male and female. The cells in green/red indicate that the food-habit constraint on the maximum/minimum consumption of that food is binding.


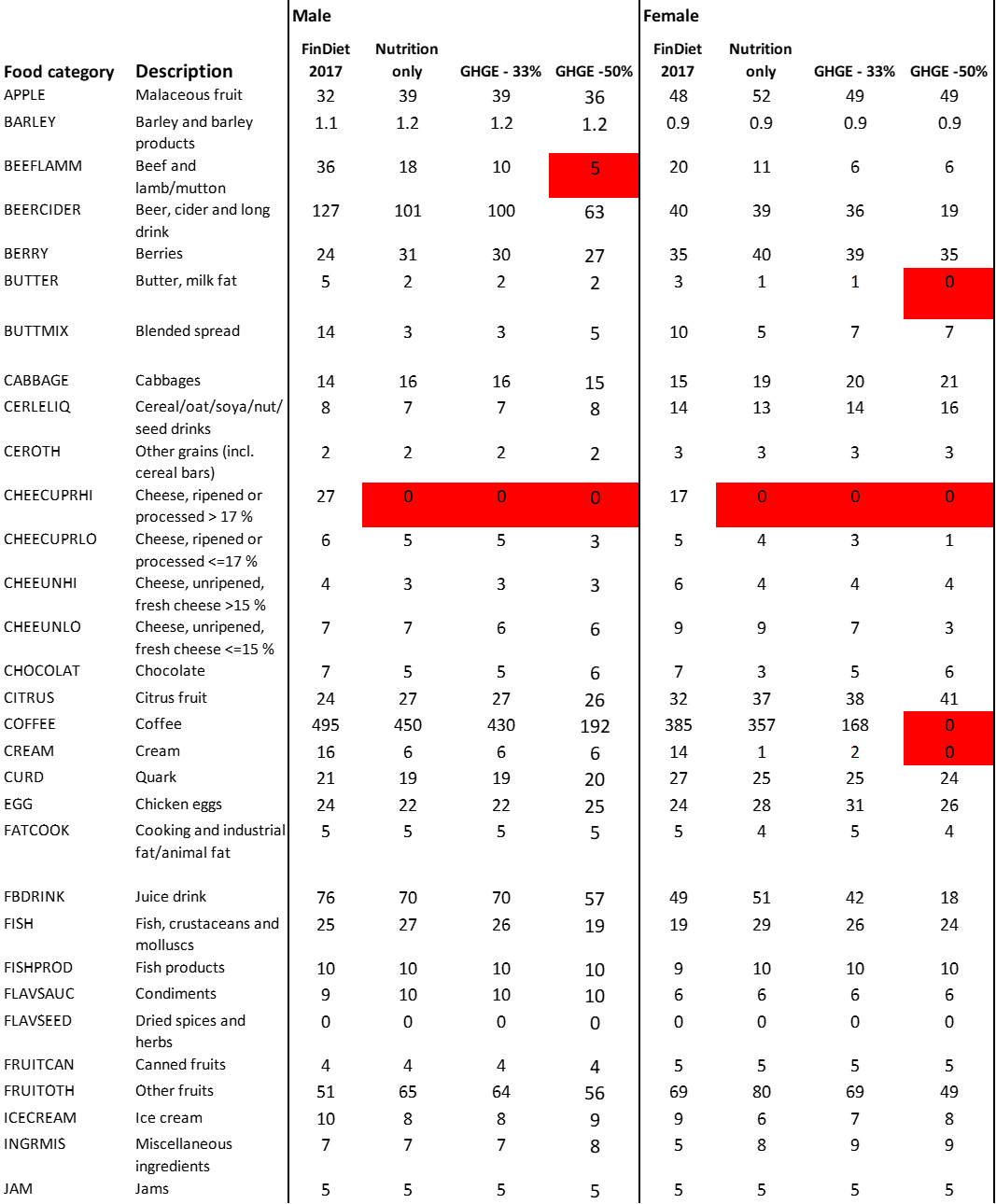


**
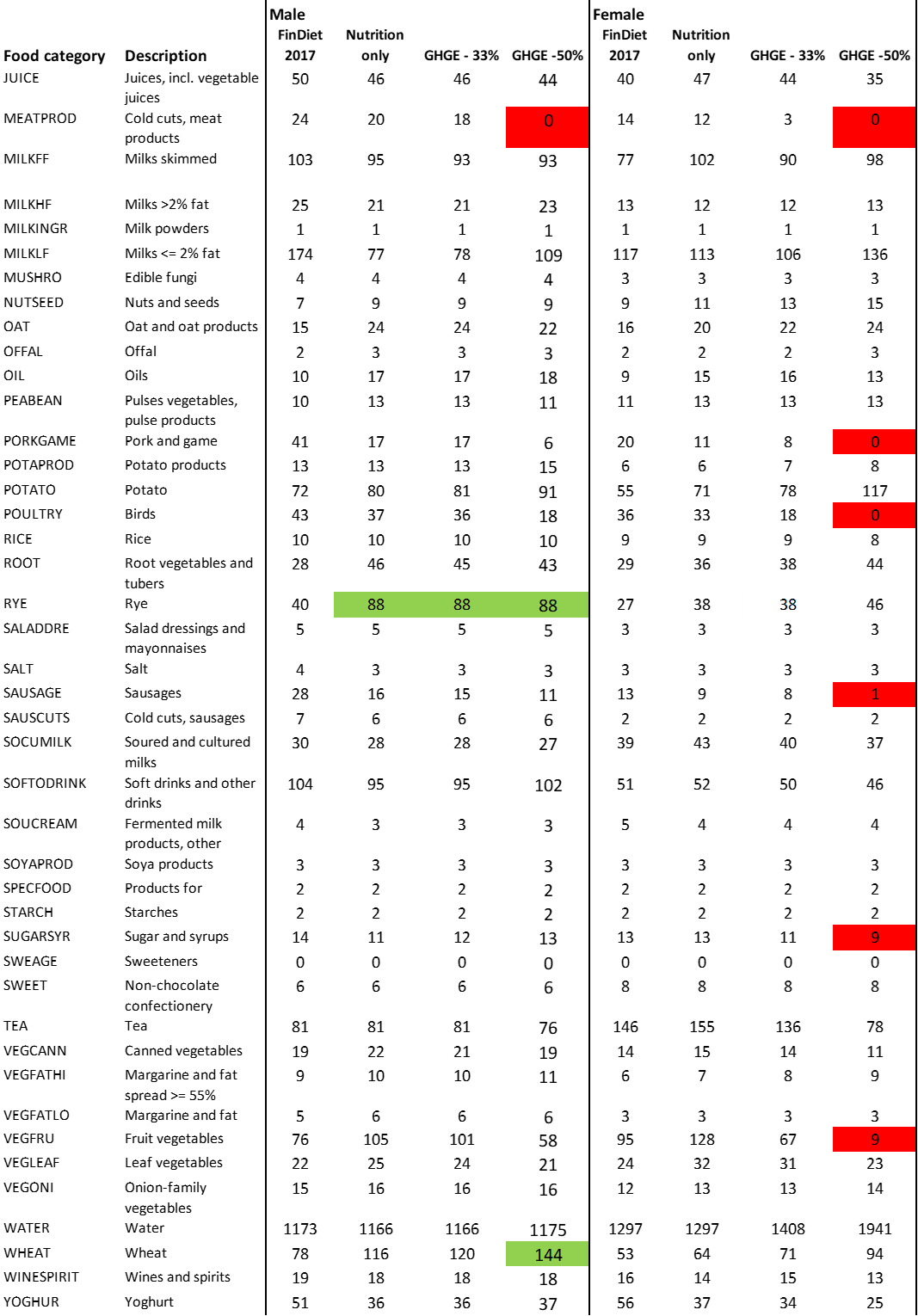
**

**Table C.2:** Initial and optimized diets (g/cap/day), adult Finnish female by educational category. The cells in green/red indicate that the food-habit constraint on the maximum/minimum consumption of that food is binding. The ingredient classes are defined in Table C.1.
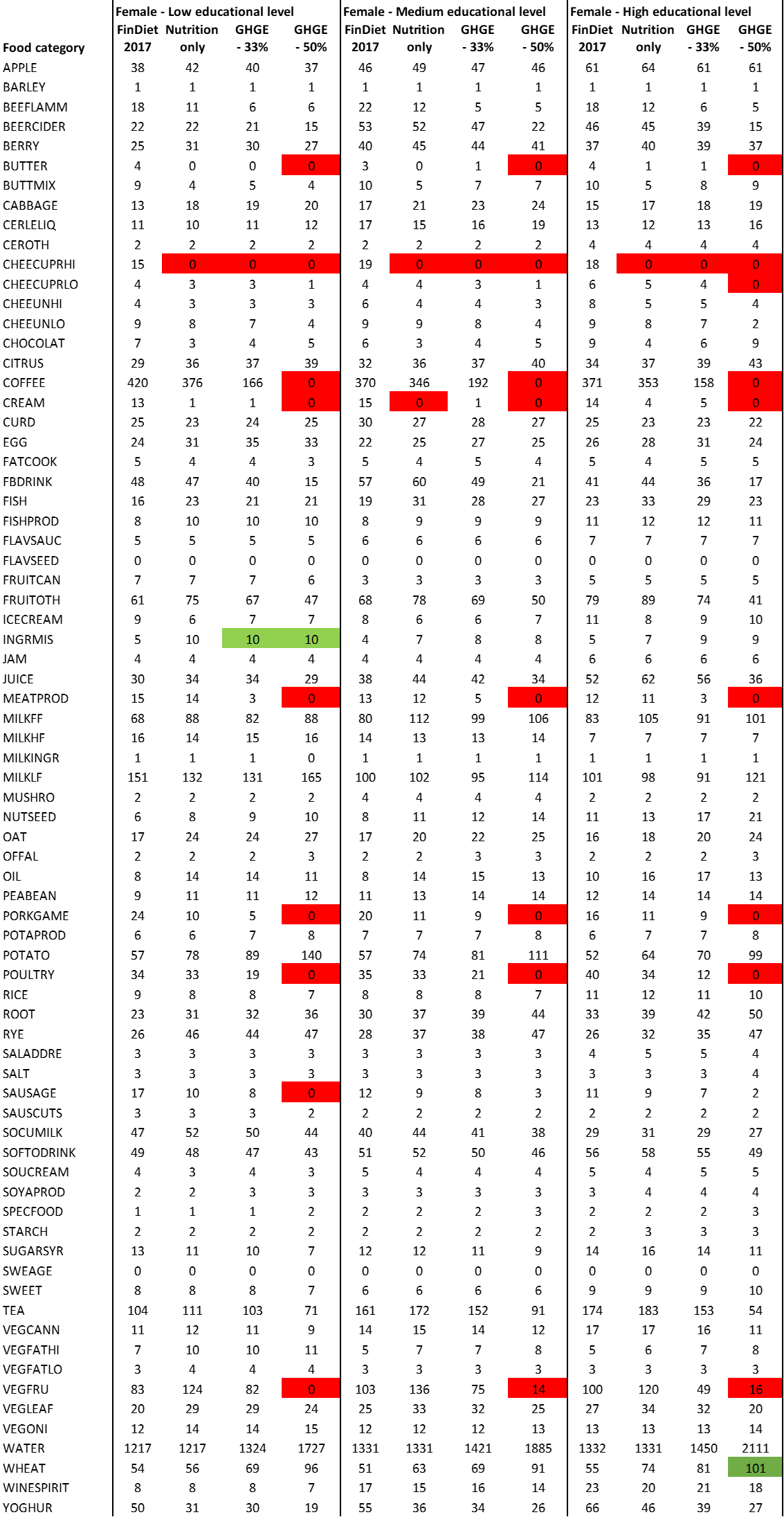


**Table C.3:** Initial and optimized diets (g/cap/day), adult Finnish male, by educational categories. The cells in green/red indicate that the food-habit constraint on the maximum/minimum consumption of that food is binding. The ingredient classes are defined in Table C.1

.
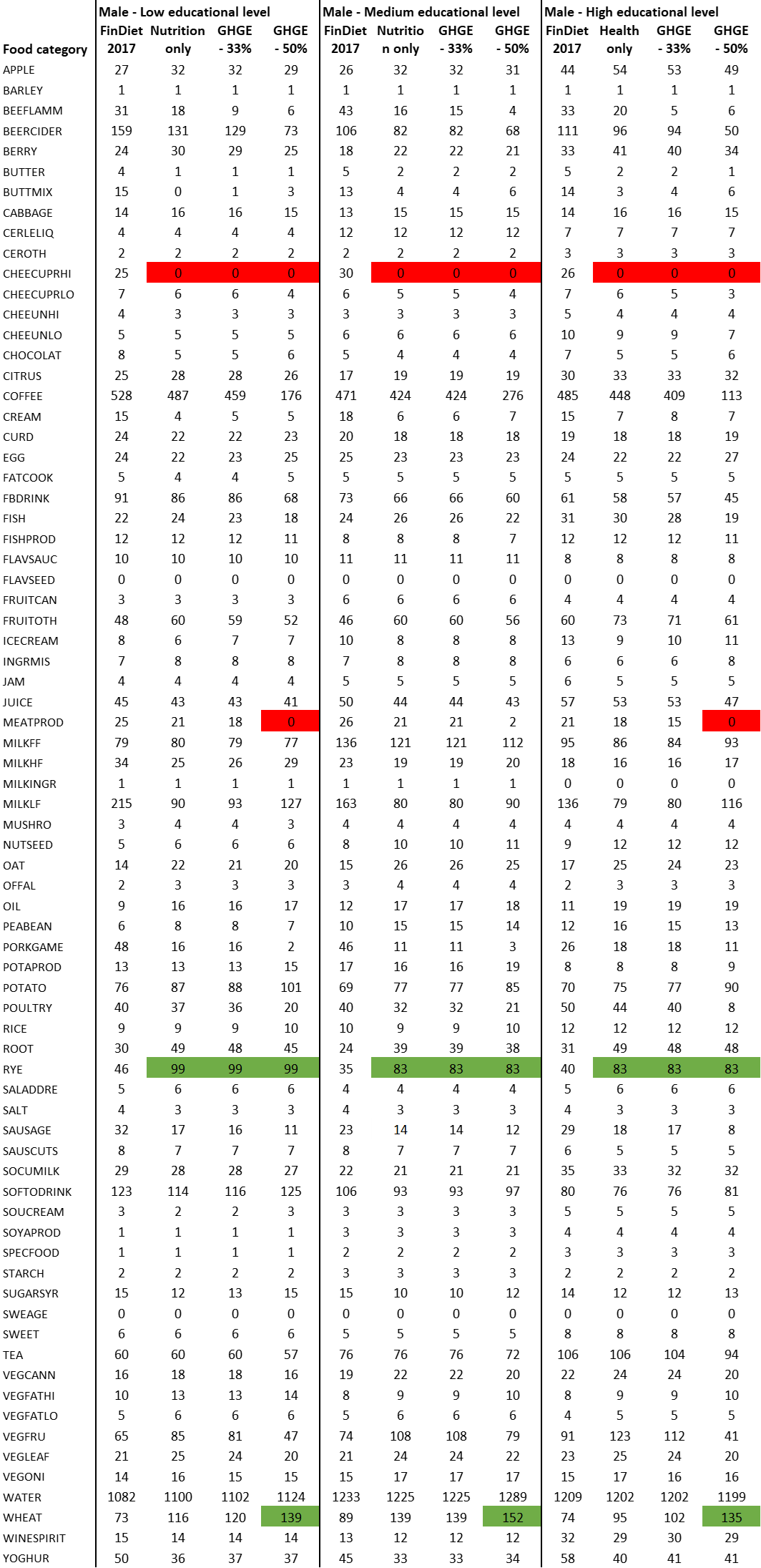


**Table C.4:** Initial and optimized diets (g/cap/day), adult Finnish female, by income quintiles. The cells in green/red indicate that the food-habit constraint on the maximum/minimum consumption of that food is binding. The ingredient classes are defined in Table C.1.


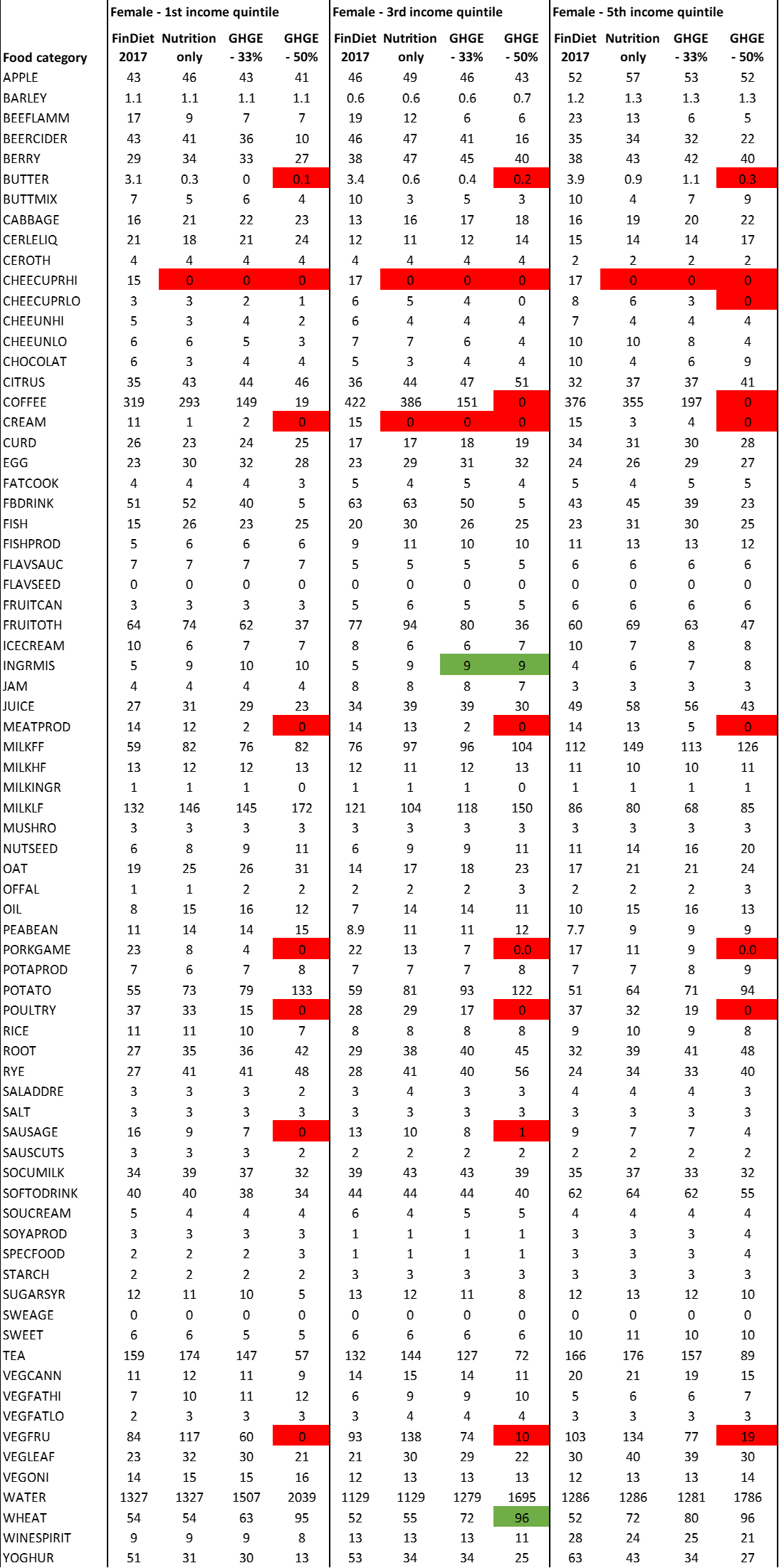


**Table C.5:** Initial and optimized diets (g/cap/day), adult Finnish male, by income quintiles. The cells in green/red indicate that the food-habit constraint on the maximum/minimum consumption of that food is binding. The ingredient classes are defined in Table C.1.


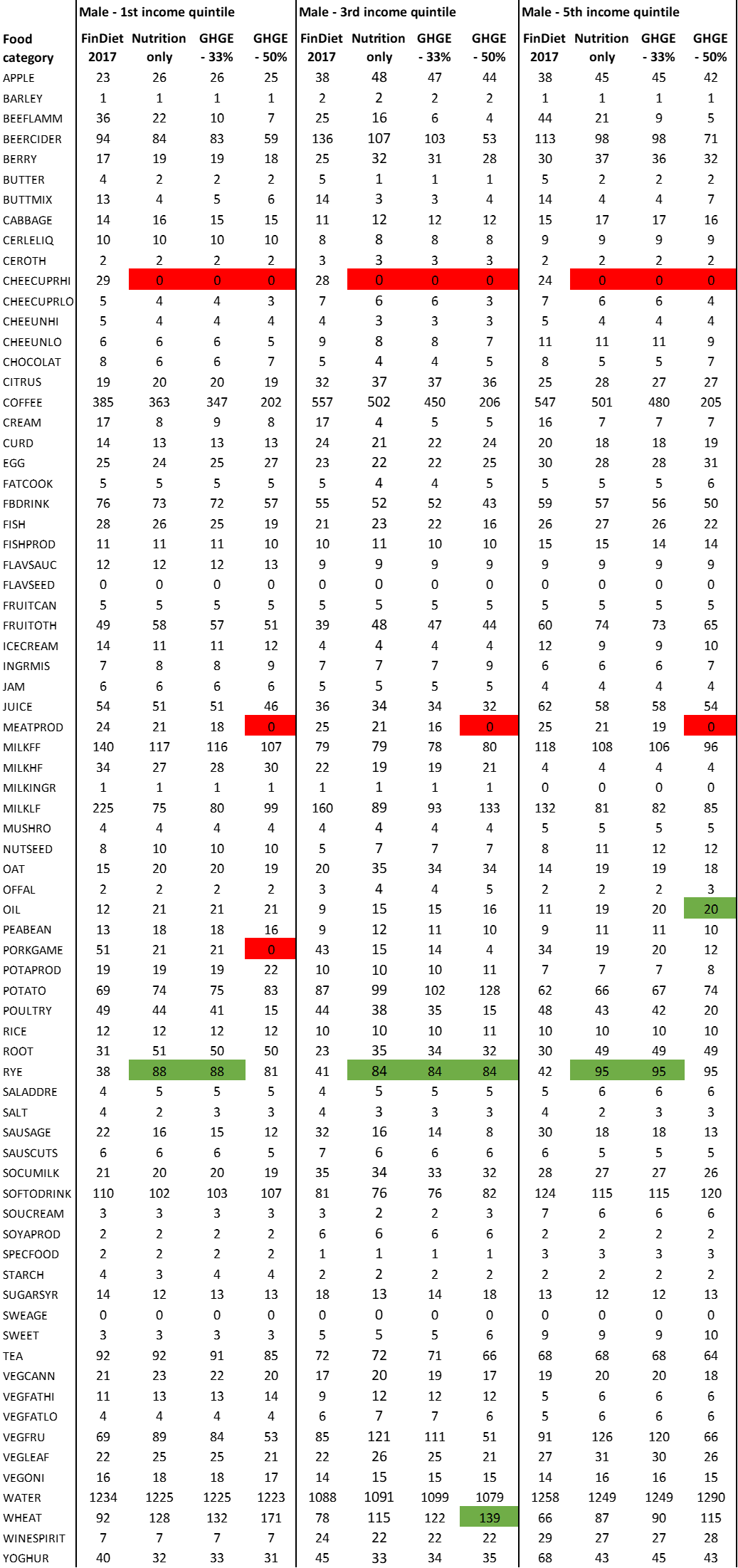


**Appendix D – Detailed nutritional and environmental properties of the baseline and simulated diets**

**Table D.1:** Nutritional and climate characteristics of the baseline and optimized diets, average adult Finnish male and female (amount/cap/day). The cells in orange indicate that the constraint is binding.


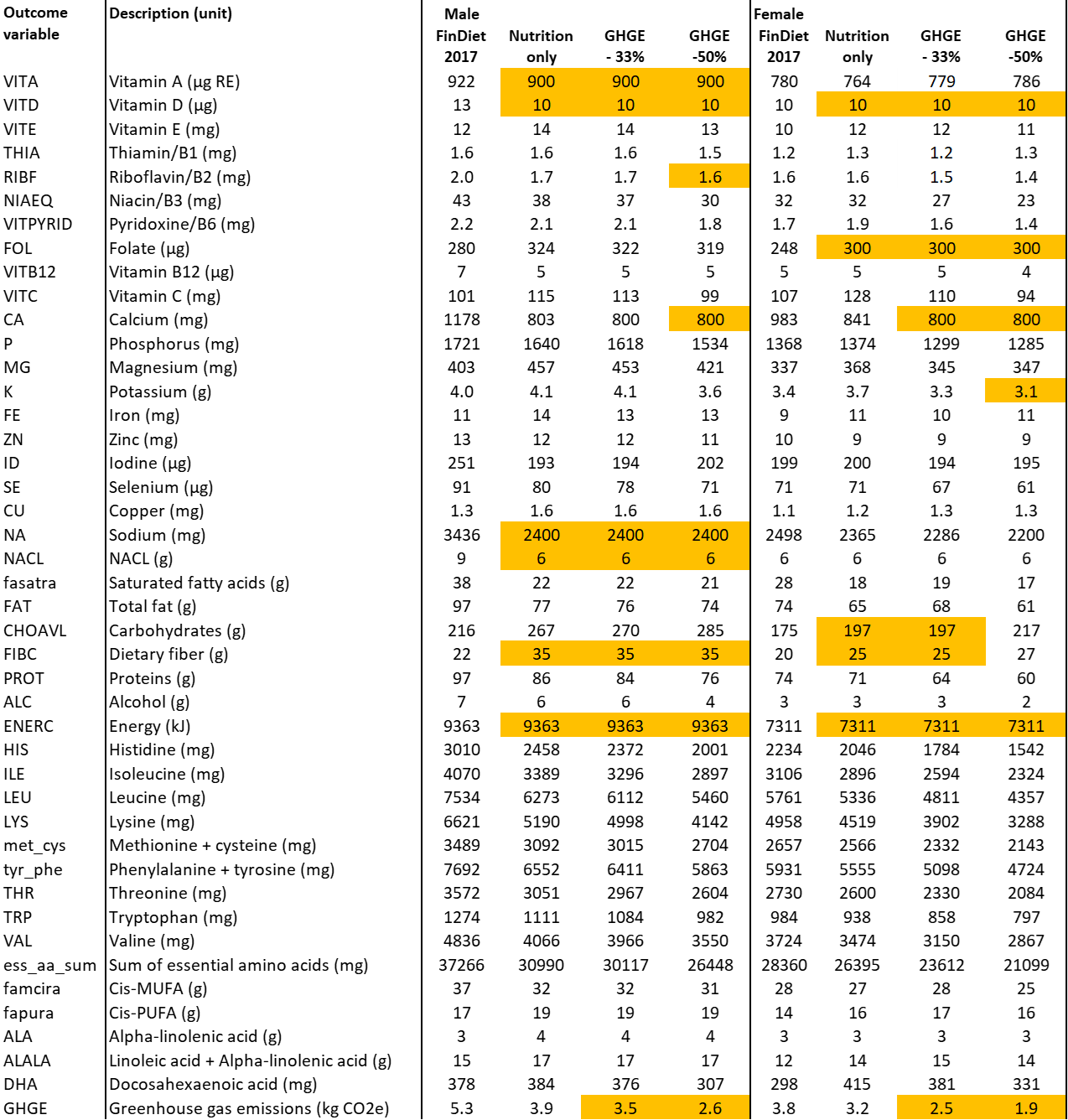


**Table D.2:** Nutritional and environmental characteristics of the baseline and optimized diets, average adult Finnish female in three educational categories. The cells in orange indicate that the constraint is binding. The outcome variables are defined in Table D.1.


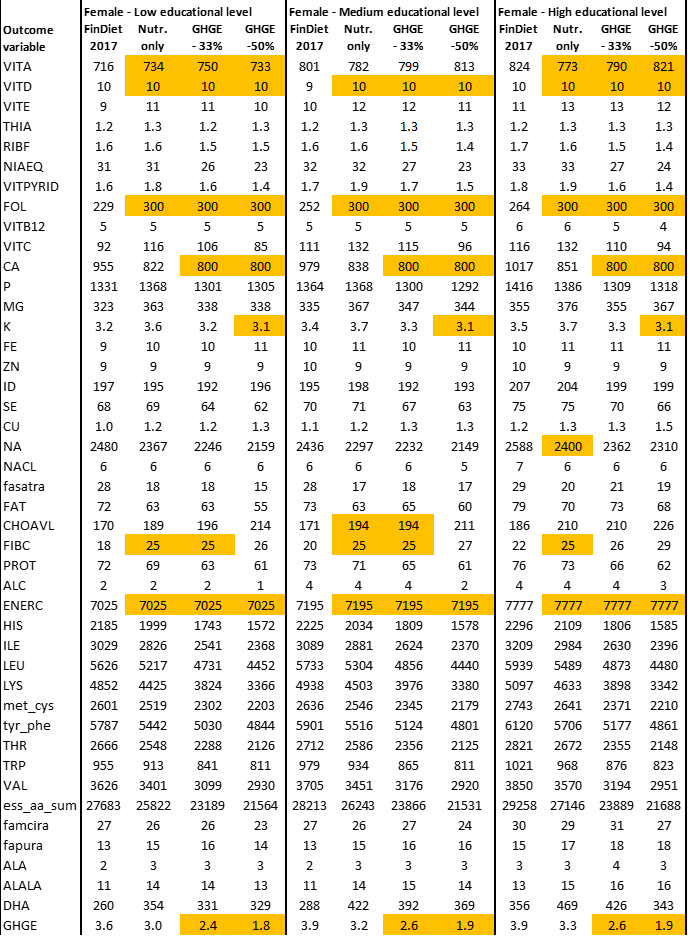


**Table D.3:** Nutritional and climate characteristics of the baseline and optimized diets, average adult Finnish male in three educational categories. The cells in orange indicate that the constraint is binding. The outcome variables are defined in Table D.1.


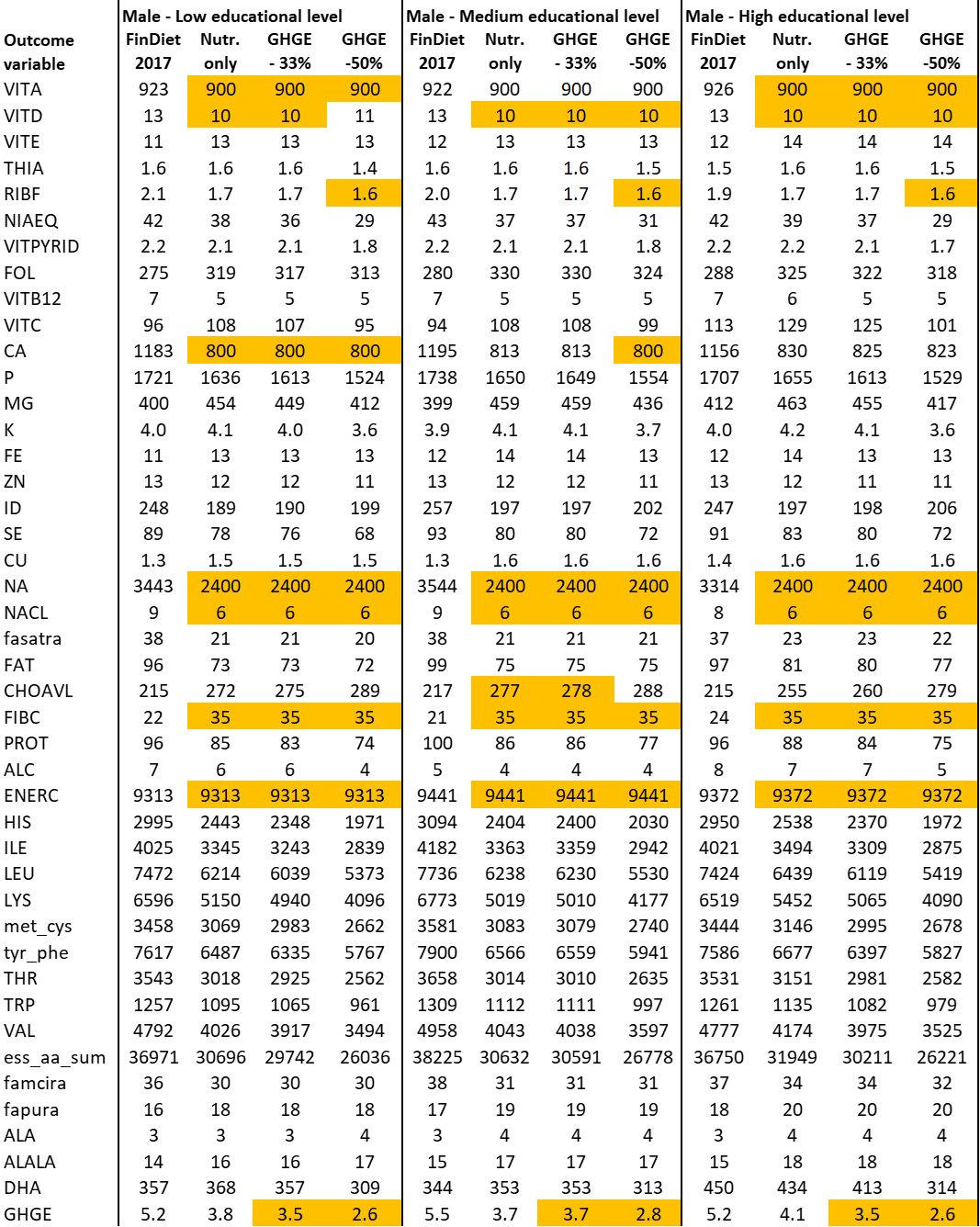


**Table D.4:** Nutritional & environmental characteristics of the baseline and optimized diets, average adult Finnish female in 1st, 3rd and 5th income quintiles. The cells in orange indicate that the constraint is binding. The outcome variables are defined in Table D.1.
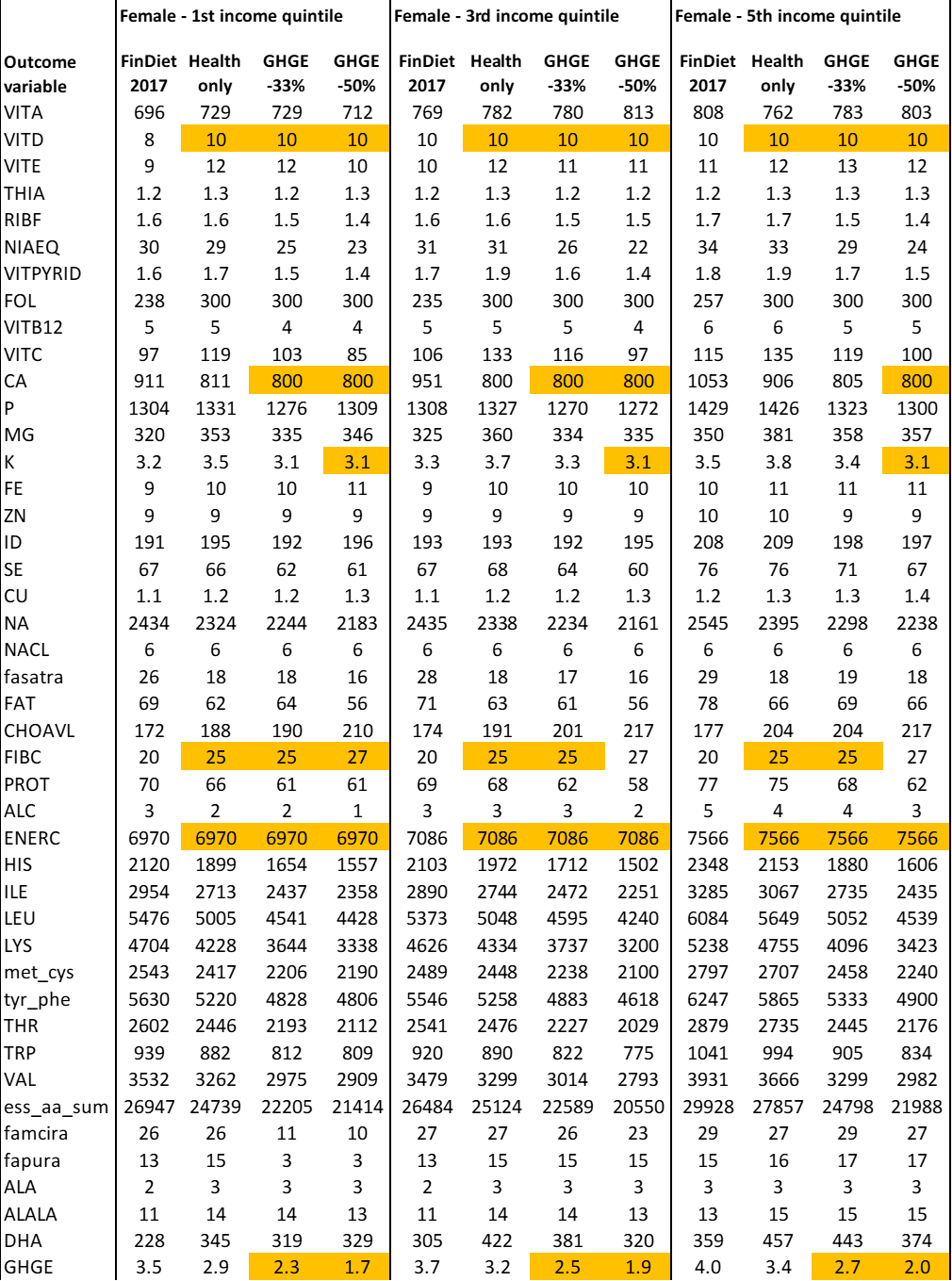


**Table D.5:** Nutritional and climate characteristics of the baseline and optimized diets, average adult Finnish male in first, third and fifth income quintiles. The cells in orange indicate that the constraint is binding. The outcome variables are defined in Table D.1.


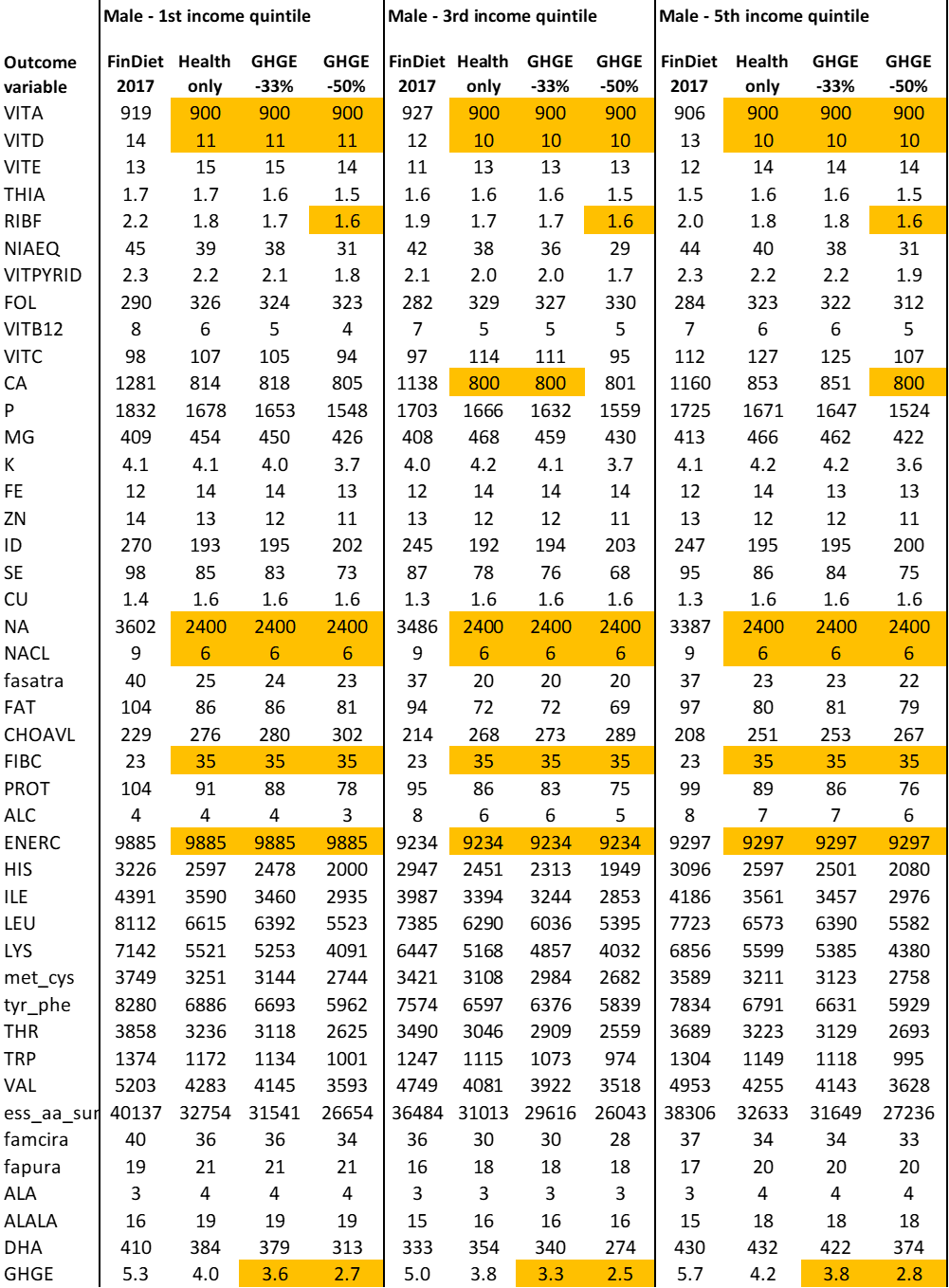

Supplement: Irz et al. supplementary material [file S1368980024000508sup001.docx]
